# Disentangling the effects of PTSD from Gulf War Illness in male veterans via a systems-wide analysis of immune cell, cytokine, and symptom measures

**DOI:** 10.1186/s40779-023-00505-4

**Published:** 2024-01-02

**Authors:** Esha Sultana, Nandan Shastry, Rishabh Kasarla, Jacob Hardy, Fanny Collado, Kristina Aenlle, Maria Abreu, Emily Sisson, Kimberly Sullivan, Nancy Klimas, Travis J. A. Craddock

**Affiliations:** 1 Institute for Neuro-Immune Medicine, Nova Southeastern University, Ft. Lauderdale-Davie, FL 33314 USA; 2 Department of Psychology and Neuroscience, Nova Southeastern University, Ft. Lauderdale-Davie, FL 33314 USA; 3 Department of Clinical Immunology, Nova Southeastern University, Ft. Lauderdale-Davie, FL 33314 USA; 4 Miami Veterans Affairs Medical Center, Miami, FL 33125 USA; 5 Department of Environmental Health, Boston University School of Public Health, Boston, MA 02118 USA; 6 Department of Computer Science, Nova Southeastern University, Ft. Lauderdale-Davie, FL 33314 USA

**Keywords:** Gulf War Illness, Post-traumatic stress disorder, Cytokine signalling, Flow cytometry, Correlation networks, Complete blood count, Subtyping, Trauma, Symptom presentation

## Abstract

**Background:**

One-third of veterans returning from the 1990–1991 Gulf War reported a myriad of symptoms including cognitive dysfunction, skin rashes, musculoskeletal discomfort, and fatigue. This symptom cluster is now referred to as Gulf War Illness (GWI). As the underlying mechanisms of GWI have yet to be fully elucidated, diagnosis and treatment are based on symptomatic presentation. One confounding factor tied to the illness is the high presence of post-traumatic stress disorder (PTSD). Previous research efforts have demonstrated that both GWI and PTSD are associated with immunological dysfunction. As such, this research endeavor aimed to provide insight into the complex relationship between GWI symptoms, cytokine presence, and immune cell populations to pinpoint the impact of PTSD on these measures in GWI.

**Methods:**

Symptom measures were gathered through the Multidimensional fatigue inventory (MFI) and 36-item short form health survey (SF-36) scales and biological measures were obtained through cytokine & cytometry analysis. Subgrouping was conducted using Davidson Trauma Scale scores and the Structured Clinical Interview for Diagnostic and statistical manual of mental disorders (DSM)-5, into GWI with high probability of PTSD symptoms (GWI_H_) and GWI with low probability of PTSD symptoms (GWI_L_). Data was analyzed using Analysis of variance (ANOVA) statistical analysis along with correlation graph analysis. We mapped correlations between immune cells and cytokine signaling measures, hormones and GWI symptom measures to identify patterns in regulation between the GWI_H_, GWI_L_, and healthy control groups.

**Results:**

GWI with comorbid PTSD symptoms resulted in poorer health outcomes compared with both Healthy control (HC) and the GWI_L_ subgroup. Significant differences were found in basophil levels of GWI compared with HC at peak exercise regardless of PTSD symptom comorbidity (ANOVA *F* = 4.7, *P* = 0.01,) indicating its potential usage as a biomarker for general GWI from control. While the unique identification of GWI with PTSD symptoms was less clear, the GWI_L_ subgroup was found to be delineated from both GWI_H_ and HC on measures of IL-15 across an exercise challenge (ANOVA *F* > 3.75, *P* < 0.03). Additional differences in natural killer (NK) cell numbers and function highlight IL-15 as a potential biomarker of GWI in the absence of PTSD symptoms.

**Conclusion:**

We conclude that disentangling GWI and PTSD by defining trauma-based subgroups may aid in the identification of unique GWI biosignatures that can help to improve diagnosis and target treatment of GWI more effectively.

**Supplementary Information:**

The online version contains supplementary material available at 10.1186/s40779-023-00505-4.

## Background

Nearly one-third of veterans returning from Operation Desert Storm/Shield during the 1990–1991 Persian Gulf War conflict reported a myriad of symptoms shortly after returning home including cognitive dysfunction, skin rashes, musculoskeletal discomfort, and fatigue [1]. This convergence of symptoms later became referred to as Gulf War Illness (GWI). To date, the underlying mechanisms of GWI disease activity have yet to be fully elucidated, and diagnosis and treatment are based on symptomatic presentation. One of the most common symptoms of GWI, impaired cognitive function, has been linked to chronic inflammation, mitochondrial dysfunction, as well as elevated oxidative stress [2]. The disruption of multiple systemic functions results in a complex clinical presentation of the illness. As a result, accepted biomarkers and mechanisms for treatment are difficult to identify, therefore, afflicted veterans are commonly diagnosed based on psychological or psychiatric evaluations [3]. One confounding factor tied to the illness is the presence of comorbidities, such as post-traumatic stress disorder (PTSD), that may influence GWI pathophysiology.

PTSD is a psychological condition that arises from exposure to trauma which results in symptoms including vivid flashbacks, frightening thoughts, avoidance of certain associated places or events, and a constant state of hyperarousal [4, 5]. These symptoms of PTSD are accompanied by negative alterations in cognition and mood as defined by the Diagnostic and Statistical Manual of Mental Disorders (DSM)-5/International Classification of Diseases-10 [6]. PTSD may also contribute to negative physical health states/symptoms such as a marked increase of musculoskeletal pain and cardio-respiratory complications [7].

It has been documented that many veterans with wartime exposures report symptoms of PTSD such as sudden and unexpected panic, disturbance of sleep, intrusive/unwanted memories and associations caused by the event, alterations in mood, general fatigue, loss of concentration, anhedonia, and a reluctance for social behavior/relationships [8, 9]. The point prevalence of combat-related PTSD reported across studies of US combat veterans ranges from about 2–17%; and lifetime prevalence about 6–31% with rates from veterans of the Vietnam War ranging from 2.2–15.2%, and among Persian Gulf War veterans of 1.9–13.2% [10]. However, when considering comorbidity, Weiner et al. [11] found PTSD prevalence rates in Gulf War veterans of 3.1% in the absence of GWI criteria and 34.6% meeting GWI criteria. A third intermediate group endorsing some combination of GWI symptoms, but not meeting full GWI criteria had a 17.8% prevalence rate of PTSD suggesting a relationship between GWI symptoms and PTSD. Investigations into the effect of PTSD on GWI symptoms in both male and female cohorts have also found an increasing severity of GWI symptoms with increasing post-traumatic stress symptoms, while still finding GWI symptoms in the absence of any post-traumatic stress symptoms [12, 13]. Despite the apparent relationship between PTSD and increased GWI severity, research efforts evaluating the impact of PTSD on GWI pathophysiology have been limited.

Previous studies examining GWI and PTSD indicated that the symptomology of both conditions strongly overlap. This overlap complicates the ability to disentangle and thus accurately diagnose GWI when comorbid with PTSD. Such complications in diagnostics may affect the potential treatment approaches clinicians employ and therefore have a direct impact on patient care and recovery. While GWI and PTSD are distinct illnesses, the complex interactions occurring due to overlapping symptomology questions whether PTSD may be modulating the nature and severity of symptoms reported by GWI confirmed veterans. Indeed, previous research has shown that GWI veterans with a confirmed PTSD diagnosis have a significantly higher likelihood of reporting abdominal pain, musculoskeletal pain, joint pain, and back pain including modulated vagal and heart-rate dynamics [14–17]. It has also been shown that PTSD may suppress innate immune activity, resulting in compromised inflammatory activity [18]. Immunological dysfunction in relation to PTSD has specifically been associated with changes in circulating cytokine levels [interleukin (IL)-1β, IL-4, IL-6, IL-8, IL-10, interferon (IFN)-γ and tumor necrosis factor (TNF)-α] and altered immune cell activity [19–23]. Impaired immune function has also been observed in GWI, with exercise accentuating the altered immune signature [24–29]. Therefore, identifying GWI veterans with and without comorbid PTSD can produce subgroups with distinct phenotypes due to specific changes in cytokine and immune cell profiles.

Of particular relevance to the study of GWI and PTSD comorbidity is the recent work showing that genetic variants in the *PON1* gene are associated with an increased rate of developing GWI after chemical exposure [30]. *PON1* encodes for serum paraoxonase 1 (PON-1) an enzyme that has multifunctional roles in various biochemical pathways such as protection against oxidative damage and lipid peroxidation, contribution to innate immunity, detoxification of reactive molecules, bioactivation of drugs, modulation of endoplasmic reticulum stress and regulation of cell proliferation/apoptosis. The variant PON1 found in GWI leads to a reduced enzymatic ability to hydrolyze organophosphate compounds, such as the nerve agent sarin and pesticide chlorpyrifos [30], and the pyridostigmine bromide (PB) pills [31] which have been associated with GWI. While the activities and isoforms of PON-1 do not appear to have any relationship to deployment in Gulf War era veterans [32], lowered PON-1 activities do appear to be a key component in the ongoing nitric oxide stress processes that accompany affective disorders, general anxiety and schizophrenia [33]. In line with this, compared with non-trauma-exposed controls, those with PTSD appear to have decreases in paraoxanase levels [34, 35]. However, compared with trauma-exposed controls who didn’t develop PTSD this does not appear to be the case [35], as not all individuals exposed to the same traumatic event develop PTSD due to pre-existing genetic, physiological, psychological and/or environmental factors making some individuals more vulnerable than others. Despite this, the high prevalence rate of PTSD noted in GWI veterans [11], and the involvement of PON-1 in both GWI and PTSD suggest comorbid interaction in their pathophysiology that needs to be addressed.

These overlaps between symptomology and pathophysiology of GWI and PTSD highlight the importance of understanding the underlying mechanisms mediating both illnesses and the implications of PTSD on GWI presentation and progression. Specifically, research efforts have proved that both GWI and PTSD are associated with immune dysfunction and teasing out contributions from PTSD can provide an improved understanding of GWI pathophysiology and progression in the absence and presence of comorbidity. This may help to stratify GWI into distinctive subtypes to improve diagnosis and target treatment more effectively, leading to improved care and better quality of life for ailing veterans. This research endeavor builds on our previous research, which involves the mapping of complex inflammatory mechanisms associated with GWI. Specifically, we aimed to achieve an understanding of the unique symptoms, cytokines, and signaling pathways characterizing the subgroups of GWI as well as distinguishing any compounding dysregulation caused by PTSD.

## Methods

### Ethics approval and consent to participate

Participants were recruited through several studies carried out at multiple institutions. Participants recruited via the Miami Veterans Affairs Medical Center (MVAMC) signed an informed consent approved by the Institutional Review Board (IRB) of the MVAMC (Protocol numbers 4987.69 and 4987.75). Participants recruited via Boston University School of Public Health signed an informed consent approved by Boston University Medical Campus IRB. Ethics review and approval for data analysis was obtained by the IRB of Nova Southeastern University (NSU) and approved by the United States Army Medical Research and Material Command (USAMRMC) Human Research Protection Office (HRPO).

### Cohort

Participants were recruited in three cohorts through both the MVAMC (2 cohorts) and through Boston University (1 cohort). The following describes the cohorts recruited from each site.

#### Miami cohorts

Two cohorts were recruited via the Miami Veterans Affairs Medical Center (MVAMC) under a Veterans Affairs Merit award [GWI: *n* = 27, healthy controls (HC): *n* = 25] and a Department of Defense (DOD) GWI Research Program award (GWI: *n* = 24, HC: *n* = 19), both of which compared male veterans meeting criteria for GWI to HC. Inclusion criteria for GWI participants were derived from Fukuda et al. [36] and consisted in identifying veterans deployed to wartime theater between August 8th, 1990, and July 31st, 1991, with one or more symptoms present for six months from at least two of the following: Fatigue, mood, and cognitive complaints, as well as musculoskeletal complaints. Participants were in good health prior to 1990 and had no current exclusionary diagnoses defined by Reeves et al. [37]. This includes exclusion of major dementias of any type and alcoholism or drug abuse, medical conditions including organ failure, rheumatologic disorders, and use of medications that affect immune function, such as steroids or immunosuppressants. Collins et al. [38] support the use of the Fukuda definition in GWI [36]. HC participants consisted of GW era veterans, both deployed and non-deployed, self-defined as healthy with no exclusionary diagnoses and sedentary (no regular exercise program, sedentary employment). All participants in the Miami cohort were subjected to a standard maximal graded exercise test to stimulate their immune response and blood samples were collected at Timepoint 0 – at rest (T0), Timepoint 1 – at peak exercise (T1) and Timepoint 2–4 h after peak exercise (T2) [25].

#### Boston cohort

The Boston cohort was recruited from the DOD funded Boston GWI Consortium (GWIC) [26] which is now included in the DOD funded Boston Biorepository, Recruitment, and Integrative Network for GWI (W81XWH-18-1-0549) [39]. The GWIC cohort recruited 269 total participants of which 25 male GW veteran’s data was shared for this study. All GWIC participants served in the 1991 Gulf War. GWI cases and controls were determined using the Kansas GWI criteria [40]. The Kansas GWI case criteria required two mild or one moderate-severe chronic symptom in at least three of six symptom domains which included fatigue/sleep, pain, neurologic/cognitive/mood, gastrointestinal, respiratory, and skin rash. This definition also excludes veterans who had one or more medical conditions that could account for their symptoms. For this study veterans were excluded if they endorsed any of the following medical conditions: diabetes, heart disease, stroke, lupus, multiple sclerosis, rheumatoid arthritis, Parkinson's disease, amyotrophic lateral sclerosis, seizure disorders, Alzheimer's disease, cancer other than skin cancer, liver disease, kidney disease, schizophrenia, or bipolar disorder. Those not meeting GWI case criteria or exclusionary criteria were considered healthy controls.

### Subgrouping procedure

#### Miami cohort

The Davidson Trauma Scale (DTS) is a 17-item self-report questionnaire of post-traumatic stress symptoms corresponding [41] to the DSM-IV symptoms of PTSD. A total score, reflecting both frequency and severity ratings for all 17 items and separate ratings for the total frequency and total severity of all 17 items, was used to interpret PTSD probability. The three clusters (Intrusiveness, Avoidance/Numbing, and Hyperarousal) were also scored separately. Following McDonald et al. [42], we applied a simple cut score at 70 for the total DTS score as it has been shown to offer optimal diagnostic accuracy, correctly classifying 90% of cases and providing an accurate estimate of PTSD population prevalence (12–13%). Those GWI subjects with DTS scores 70 and above were considered as probable PTSD positive with GWI with high probability of PTSD symptoms (GWI_H_), while those below 70 were considered as probable PTSD negative with GWI with low probability of PTSD symptoms (GWI_L_).

#### Boston cohort

The Clinically Administered PTSD Scale for DSM-5 (CAPS-5) is a structured clinical interview that includes 30 questions that assess current and lifetime PTSD diagnosis [43]. It includes questions from the DSM-5 PTSD symptoms and requires a traumatic event that symptoms are assessed in relation to PTSD severity, dissociative symptoms as well as impact on work and recreational functioning. The Structured Clinical Interview for DSM-5 (SCID-5) is a semi-structured interview for making the major DSM-5 diagnoses [44]. The SCID is broken down into separate modules corresponding to categories of diagnoses. A diagnosis of PTSD was made following the PTSD diagnostic algorithm and used to assign subjects to the GWI_H_ and GWI_L_ groups.

### Symptom measures

All participants from both the Boston and Miami sites received a physical examination and medical history including the GWI symptom checklist as per the case definition. Symptom questionnaires included the Multidimensional Fatigue Inventory (MFI) [45], a 20-item self-report instrument designed to measure fatigue with five resulting composite scores and the RAND Medical Outcomes Study 36-item short-form survey (SF-36) [46, 47], which assesses health-related quality of life with eight resulting composite scores. The PTSD status of the Miami cohort was evaluated with the DTS [41], a self-rating measurement of the frequency and severity of PTSD symptoms in three clusters: Intrusion, avoidance, and hyperarousal. The Boston cohort received a clinical psychiatric interview that included the CAPS-5 [43] and the SCID [44] to identify exclusionary psychiatric diagnoses (bipolar disorder, schizophrenia) and to determine psychiatric diagnoses including PTSD.

### Complete blood count (CBC)

Whole blood collected in K2-ethylenediaminetetraacetic acid (EDTA) blood tubes (B-D-Biosciences, San Jose, CA, USA) was measured using the Beckman Coulter LH500 (Beckman Coulter Inc, Brea, CA, USA) to obtain complete blood count with differentials.

### Human cytokine analysis

Cytokine analysis was performed using Quansys chemiluminescent assays (Quansys Biosciences, Logan, UT, USA). The Quansys Imager, driven by an 8.4-megapixel Canon 20D digital SLR camera, supports 96 well plate based chemiluminescent imaging. The Q-Plex™ Human Cytokine—Screen is a quantitative enzyme-linked immunosorbent assay (ELISA)-based test where distinct capture antibodies have been absorbed to each well of a 96-well plate in a defined array. Comparable custom 16- and 18-multiplex assays were used in across the studies.

The 16-multiplex panel includes TNF-α, TNF-β, IL-1α, IL-1β, IL-6, IFN-γ, IL-12, IL-2, IL-15, IL-8, IL-5, IL-17, IL-23, IL-10, and IL-13 [48]. For the standard curves, we used the second order (k = 2) polynomial regression model (parabolic curve), Y = b_0_ + b_1_X + b_2_X2… + b_k_Xk, where Y is the predicted outcome value for the polynomial model with regression coefficients b_1_ to k for each degree and y intercept b_0_. Quadruplicate determinations were made, i.e., each sample was run in duplicate in two separate assays.

The 18-multiplex panel includes TNF-α, TNF-β, IL-1α, IL-1β, IL-6, IFN-γ, IL-12, IL-2, IL-15, IL-8, IL-5, IL-17, IL-23, IL-10, IL-13, TNF-RI, and TNF-RII. Briefly, plasma samples are thawed at 4 °C overnight. Samples are plated in duplicate following manufacturers protocol. The plates were read at 270 s of exposure time using the Q-view Imager LS (Quansys Biosciences, Logan, UT, USA). Individual cytokine concentrations were obtained using the image analysis software Q-view (Quansys Biosciences, Logan, UT, USA). Sample concentrations were calculated from standard curves created by a five-parameter logistic regression with 1/y weighting. The average value from each duplicate was then used for subsequent analyses.

To account for 16-plex and 18-plex differences, data were separated by 16 and 18 plex and standardized by z-scoring. The two data sets were then combined and reverse z-scored using the mean and standard deviation of the 18-plex dataset to scale the 16-plex data to the 18-plex set. Following this the dataset was normalized using min–max normalization so the final data values ranged between 0 and 1.

### Flow cytometry

Flow cytometry was performed on each patient and healthy control subject sample to determine various immune cell populations including T cells, lymphocyte subsets and Natural Killer (NK) cells abundance using a Beckman Coulter FC500 Flow Cytometer (Beckman Coulter Inc, Brea, CA, USA). Whole blood samples were stained in multi-color combinations, with the appropriate concentrations of antibodies (Beckman Coulter Inc, Brea, CA, USA), erythrocytes lysed, and the cells fixed with the Optilyse C Lysing Solution (Beckman Coulter Inc, Brea, CA, USA). Lymphocyte, monocyte, and granulocyte populations were determined using light scatter and backgating on fluorescence for the markers and negative population [49, 50]. Isotype controls were used to determine the background caused by nonspecific antibody binding. A list of antibodies and fluorochrome conjugate cocktails used is provided in Additional file 1: Table S1. Spectral compensation was established daily. Values normalized to healthy controls are presented in the results. Copy of data output showing gating and graphs for various markers analyzed along with isotype controls is presented as supplementary data in Additional file 2: Flow cytometry gating strategy.

### NK cell cytotoxicity

The bioassay for NK cell cytotoxicity was performed using whole blood within 8 h of collection in a chromium release assay as previously described [50]. The NK sensitive erythroleukemic K562 cell line was used as the target cell. The assay was done in triplicate at four target-to-effector cell ratios with 4 h incubation. The % activity at each target-to-effector ratio and number of cluster differentiation (CD)3^−^CD56^+^ (NK) cells per unit of blood was used to express the results as % cytotoxicity at a target-to-effector cell ratio of 1:1.

### Hormone analysis

Serum samples were analyzed for concentrations of testosterone by immunoelectro-chemiluminescence assays on a Roche Cobas 6000 analyzer (Roche Diagnostics, Basel, Switzerland), following all manufacturer's instructions for instrument maintenance and assay calibration and test procedures with interassay % (coefficients of variation) CVs that are consistently < 4%. Salivary cortisol was determined by immunoassay using the Salimetrics high sensitivity kit (State College, PA, USA).

### Statistical analysis

Following group assignment, values were compared using ANOVA as the groups were unbalanced in size with unequal variances [51–55﻿]. All measures from the omnibus ANOVA test with a *P-*value of less than 0.05 were chosen for post-hoc analysis. Following ANOVA, a Games-Howell post-hoc analysis was performed on significant omnibus tests to determine pairwise differences with a *P-*value of less than 0.05 taken as significant [56﻿]. The effect size of the difference between groups for each measure was also estimated using the corrected Hedges g [57]. The corrected Hedges g was chosen as it gives better estimates for small sample size and is corrected to account for bias as an estimator for the population effect size. Effect sizes were interpreted in the following ranges [58﻿]: Negligible, lower than 0.01, very exceedingly small 0.01–0.20, small 0.20–0.50, medium 0.50–0.80, large 0.80–1.20, very exceptionally large 1.20–2.00, and huge 2.00 or higher. While a *P-*value can inform the reader whether an effect exists, the *P-*value will not reveal the size of the effect. In reporting and interpreting these studies, both the substantive significance (effect size) and statistical significance (*P-*value) are essential results to be reported [59]. As such, throughout the description of the results the small, medium, large, huge designations refer to the calculated Hedges’ g effect size values. All calculations were performed using MATLAB (version: 9.12.0, R2022a, The MathWorks Inc., Natick, MA, USA). Statistical analysis data can be found in Additional file 3: Complete Data and Analysis.

Correlation graphs were constructed to express the statistical relationship correlating immune cell abundance, cytokine concentration, blood counts and symptom severities and gauge their influence upon one another for each condition at each timepoint. Correlations were assessed by use of a Spearman correlation coefficient-ρ, which stands as a measure of association that assesses the extent of change between two variables (such as cytokine population and symptom severity) revealing any monotonic relationships. To account for the multiple correlation tests the relationships were filtered using the Storey corrected *P-*value, *q* [60﻿]. Correlations were taken as significant for Storey’s *q* < 0.05. The resulting graphs were then compared via the graph edit distance (GED) measure defined as the minimum amount of edit operations needed to transform one graph into another. Changes can include deletions, insertions, and substitutions of correlations that make up the graph [61, 62]. GED measures have allowed similarity comparisons to occur between graphs with relatively similar information. Roughly, the measure itself dictates how many edits to the first graph need to be performed to obtain the second graph. This yields a number that quantifies the similarity or dissimilarity of the pair of graphs. A higher number would indicate a larger number of edits and thus, a lower similarity. Whereas a lower number would indicate that there were fewer changes needed to be made thus, a higher similarity between graphs [63, 64]. Correlation analysis data can be found in Additional file 3: Complete data and analysis.

## Results

### Demographics

The total cohort used in this study was comprised of 120 male veterans recruited through the Miami and Boston based sites. For the entire cohort, the average age was (46.0 ± 7.0) years, with an average body mass index (BMI) of (30.29 ± 4.80) kg/m^2^. The sample consisted of individuals of Asian (*n* = 2, 1.7%), Black (*n* = 24, 20.0%), White Hispanic (*n* = 49, 40.8%), White (*n* = 41, 34.2%), and other (*n* = 4, 3.3%) decent. The full demographics of the participant populations with comparison statistics between groups can be found in Additional file 1: Table S2.

The full sample included male veterans (*n* = 76), meeting the criteria for GWI and HC (*n* = 44). We further subdivided the GWI sample into two groups based on their PTSD symptoms. The group of GWI subjects with DTS scores above 70 and positive PTSD SCID evaluation were considered to have a high probability of having PTSD and related symptoms and were denoted as GWI_H_ (*n* = 47), while the remainder had a low probability of having PTSD and related symptoms and were labeled as GWI_L_ (*n* = 29). All HC had low probability of having PTSD.

Statistical comparisons (Additional file 1: Table S2) were made between both total GWI and HC groups (*P*_2_), as well as between the GWI_H_, GWI_L_, and HC groups (*P*_3_) using ANOVA for continuous variables and the *χ*^*2*^ test for categorical variables. Marginally significant differences (*P* < 0.10) were found in age and BMI between HC and GWI with the GWI group being older and having a higher BMI. In addition, a statistically different breakdown in racial demographics (*P* = 0.036) was observed driven primarily by a much higher percentage of White Hispanics in the control group owing to the demographics of the Miami cohort. However, after subtyping based on the DTS no statistical differences were found in age, BMI, racial representation, or average number of years in school across controls and GWI subtypes. As most of the statistical analysis was performed on the subtyped GWI groups, these differences between the GWI and HC group were deemed to not affect the overall results of the study.

### Symptom measures

**Fig. 1:**
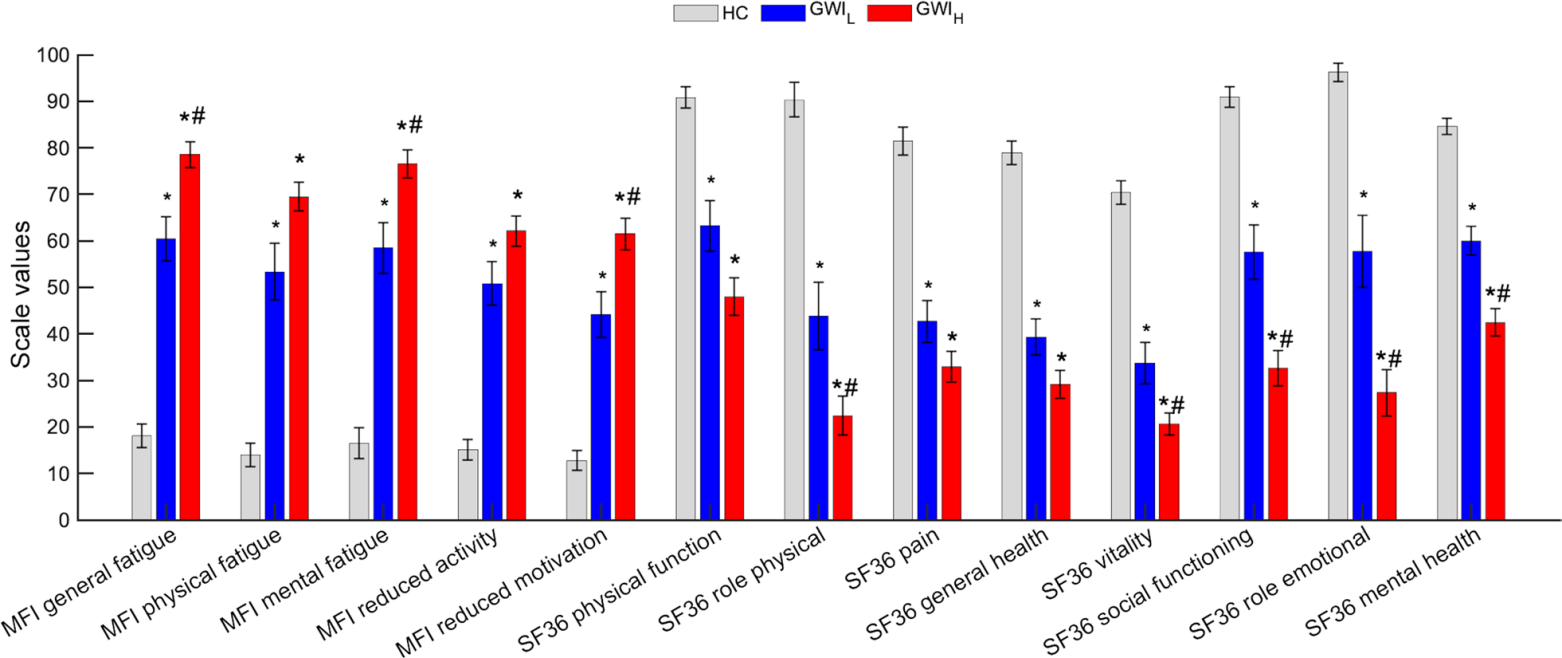
Comparison of symptom scales between trauma defined GWI groups and controls from the Miami and Boston cohorts. Standard error of the mean error bars. *P < 0.05 as compared with HC, and ^#^*P* < 0.05 as compared to GWI_L_ via Games-Howell post-hoc analysis. Higher SF36 values indicates better health. GWI_H_ Gulf War Illness with high probability of post-traumatic stress disorder (PTSD) symptoms, GWI_L_ Gulf War Illness with low probability of PTSD symptoms, HC healthy control, MFI multidimensional fatigue inventory, SF36 36-item short form healthy survey

Comparison of the MFI, SF-36, and DTS symptom scales yielded results analogous to our previous work with a smaller male cohort [12] (Additional file 1: Table S3 and Fig. 1). GWI_L_ and GWI_H_ both presented significantly worse symptom measures compared to HC (*P* ≤ 0.001), with GWI_H_ having significantly worse symptoms compared to GWI_L_ in MFI general fatigue (GWI_L_: 60.42 ± 4.71, GWI_H_: 78.53 ± 2.73, *P* = 0.006), mental fatigue (GWI_L_: 58.48 ± 5.42, GWI_H_: 76.55 ± 2.94, *P* = 0.015) and reduced motivation (GWI_L_: 44.12 ± 4.91, GWI_H_: 61.45 ± 3.46, *P* = 0.018), and SF-36 role limitations due to physical problems (GWI_L_: 43.89 ± 7.28, GWI_H_: 22.42 ± 4.12, *P* = 0.037), vitality (GWI_L_: 33.75 ± 4.45, GWI_H_: 20.68 ± 2.36, *P* = 0.039), social functioning (GWI_L_: 57.59 ± 5.90, GWI_H_: 32.61 ± 3.76, *P* = 0.004), role limitations due to emotional problems (GWI_L_: 57.74 ± 7.79, GWI_H_: 27.36 ± 4.97, *P* = 0.007) and mental health (GWI_L_: 60.00 ± 3.08, GWI_H_: 42.47 ± 2.94, *P* = 0.001). GWI, with or without trauma, had a large to huge effect size (as defined in the methods) on all SF-36 and MFI scores for each subtype compared to HC (g ≥ 1.25). Compared to GWI_L_ the PTSD symptoms of GWI_H_ worsened all SF-36 measures by a medium amount (0.50 ≤ g < 0.80) except for social functioning (g = 0.89), role limitations due to emotional problems (g = 0.81), and mental health (g = 0.92), which were affected by a large amount. For the MFI measures, the PTSD symptoms of GWI_H_ had a medium negative effect (0.50 ≤ g < 0.80) on all measures compared to GWI_L_, except for reduced activity (g = 0.48) and general fatigue (g = 0.83), which were affected by a small and large amount, respectively.

### Biological measures

**Fig. 2:**
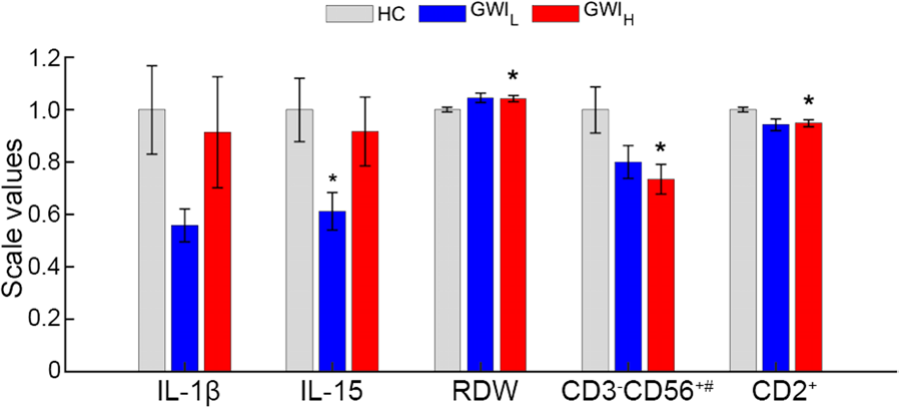
Significant comparisons of hormones, cytokines, complete blood count and flow cytometry measures at rest between trauma defined GWI groups and controls from the Miami and Boston cohorts. Standard error of the mean error bars. *P < 0.05 as compared to HC, and ^#^*P* < 0.05 as compared to GWI_L_ via Games-Howell post-hoc analysis. All values scaled to HC. GWI_H_ Gulf War Illness with high probability of post-traumatic stress disorder (PTSD) symptoms, GWI_L_ Gulf War Illness with low probability of PTSD symptoms, HC healthy control, MFI multidimensional fatigue inventory, SF36 36-item short form healthy survey

At rest, the combined Miami and Boston cohorts were compared between GWI_H_, GWI_L_, and HC groups. Significant differences between groups were shown in cytokine, CBC, and flow cytometry measures (Additional file 1: Table S4 and Fig. 2). The GWI_L_ group showed a lower concentration of IL-1β (HC: 0.14 ± 0.02, GWI_L_: 0.08 ± 0.01, GWI_H_: 0.13 ± 0.03) with a small effect size (g < 0.49) however this did not reach the level of significance (*P* > 0.057). The GWI_L_ group also showed lower IL-15 compared to HC with a significant medium effect size (HC: 0.24 ± 0.03, GWI_L_: 0.15 ± 0.02, *P* = 0.026, g = 0.58), but no significant change from GWI_H_ (GWI_H_: 0.22 ± 0.03, *P* = 0.12) despite a small effect decrease (g = 0.40). GWI_H_ showed no difference from HC in IL-15. GWI_H_ was found to have a medium effect size with lower CD3^−^CD56^+^ cell number compared to HC (HC: 201.48 ± 17.94, GWI_H_: 148.00 ± 11.44, *P* = 0.036, g = 0.53), with no significant difference from GWI_L_ (GWI_L_: 161.24 ± 12.47, *P* = 0.718, g = 0.18). GWI_L_ was shown to have a small effect size with lower CD3^−^CD56^+^ cell number compared to control, but this did not reach the level of statistical significance (*P* = 0.164, g = 0.39). GWI subtypes were found to have a higher red blood cell distribution width (RDW) compared to HC at a medium effect size (HC: 12.74 ± 0.12, GWI_L_: 13.33 ± 0.24, GWI_H_: 13.29 ± 0.16, g = 0.57), however only GWI_H_ was found to reach the level of significance (*P* = 0.018). There was no effect observed for RDW between GWI_H_ and GWI_L_ (*P* = 0.990, g = 0.03)_._ Similarly, both GWI subgroups showed a medium effect with a significantly lower percentage of CD2^+^ levels compared to HC (HC: 82.76 ± 0.71, GWI_L_: 78.05 ± 1.82, GWI_H_: 78.54 ± 1.10, g ≥ 0.65), but again only GWI_H_ reached the level of significance (*P* = 0.006), and no difference was observed between GWI subgroups (*P* = 0.971, g = 0.06).

**Fig. 3:**
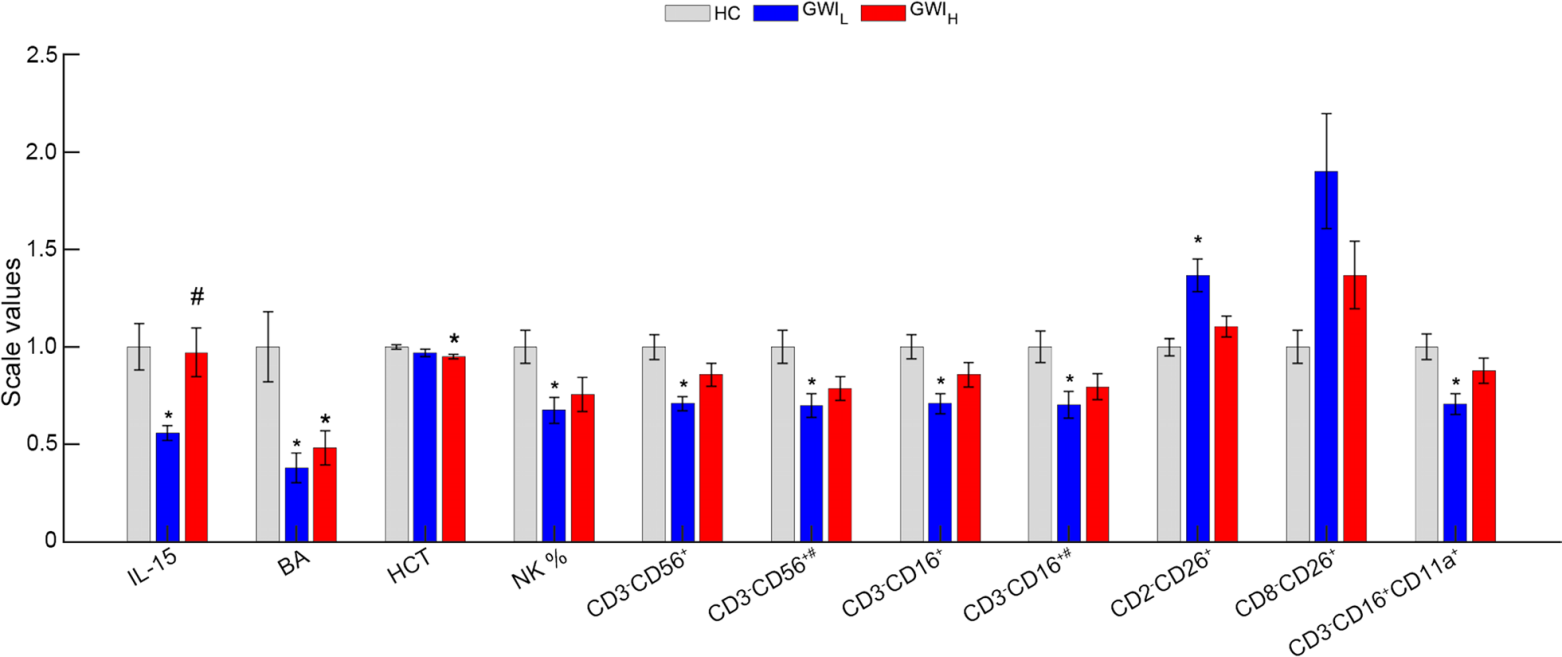
Significant comparisons of hormones, cytokines, complete blood count and flow cytometry measures at peak exercise between trauma defined GWI groups and controls from the Miami cohort. Standard error of the mean error bars. *P < 0.05 as compared to HC, and ^#^*P* < 0.05 as compared to GWI_L_ via Games-Howell post-hoc analysis. All values scaled to HC. GWI_H_ Gulf War Illness with high probability of post-traumatic stress disorder (PTSD) symptoms, GWI_L_ Gulf War Illness with low probability of PTSD symptoms, HC healthy control, MFI multidimensional fatigue inventory, SF36 36-item short form healthy survey

As only the Miami cohort underwent a graded exercise challenge, only the Miami cohort was compared between GWI_H_, GWI_L_, and HC groups at peak exercise (Additional file 1: Table S5 and Fig. 3). Similar to the at rest condition, IL-15 levels for GWI_L_ are significantly lower compared to HC with a medium effect size (HC: 0.30 ± 0.04, GWI_L_: 0.17 ± 0.01, *P* = 0.005, g = 0.66), but also at a significantly lower level with medium effect compared to GWI_H_ (GWI_H_: 0.29 ± 0.04, *P* = 0.038, g = 0.59), with no difference between GWI_H_ and HC (*P* = 0.988, g = 0.04). Both GWI subgroups showed significantly less basophils (BA) %, all at a medium level effect and lower than HC (HC: 0.40 ± 0.07, GWI_L_: 0.15 ± 0.03, GWI_H_: 0.19 ± 0.03, *P* < 0.040, g ≥ 0.52). Additionally, GWI_H_ showed a significantly lower hematocrit (HCT) than HC, at a medium effect size (HC: 47.23 ± 0.58, GWI_H_: 44.88 ± 0.48, *P* < 0.016, g = 0.64). In measures of NK % activity, and all CD3- cell type measures GWI_L_ was found to be significantly reduced by a medium to large amount compared to HC (*P* ≤ 0.043, g ≥ 0.59). While a small effect decrease for GWI_H_ compared to HC was also observed for these measures (g ≤ 0.43), none reached the level of significance (*P* ≥ 0.206). GWI_L_ was shown to have significantly higher CD2^+^CD26^+^ (%) with a large effect over HC (HC: 35.94 ± 1.58, GWI_L_: 49.12 ± 3.05, *P* = 0.013, g = 1.04), and a non-significant but medium effect over GWI_H_ (GWI_L_: 39.64 ± 1.98, *P* = 0.113, g = 0.64). While the omnibus measure of CD8^+^CD26^+^ (%) was found to be significant, no pairwise group differences were found at the level of significance despite the large increase seen in GWI_L_ compared to HC (HC: 8.38 ± 0.70, GWI_L_: 15.92 ± 2.47, *P* = 0.075, g = 0.92).

**Fig. 4:**
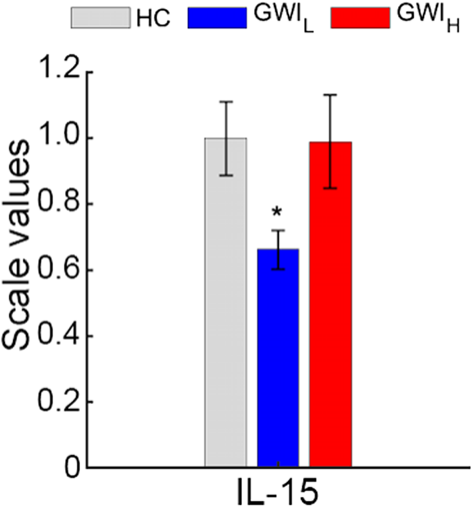
Significant comparisons of hormones, cytokines, complete blood count and flow cytometry measures 4 h post peak exercise between trauma defined GWI groups and controls from the Miami cohort. Standard error of the mean error bars. *P < 0.05 as compared to HC via Games-Howell post-hoc analysis. All values scaled to HC. GWI_H_ Gulf War Illness with high probability of post-traumatic stress disorder (PTSD) symptoms, GWI_L_ Gulf War Illness with low probability of PTSD symptoms, HC healthy control, MFI multidimensional fatigue inventory, SF36 36-item short form healthy survey

The Miami cohort was also compared between GWI_H_, GWI_L_, and HC groups for the period 4 h post exercise (Additional file 1: Table S6 and Fig. 4). Of all the measures only IL-15 was found to be significantly reduced in GWI_L_ with a medium effect size compared to HC (HC: 0.22 ± 0.02, GWI_L_: 0.14 ± 0.01, *P* = 0.044, g = 0.52). While no significant difference was found between GWI_H_ and GWI_L_, there was a small effect difference (GWI_H_: 0.22 ± 0.03, *P* = 0.215, g = 0.40), and no difference between GWI_H_ and HC (*P* = 0.999, g = 0.01).

### Correlation analysis

The GED analysis across subgroups was used to understand the differences and similarities between hormone, immune and symptoms measures, by determining the minimum number of modifications required to transform one correlation graph into another. Additional file 1: Figs. S1-S9 show the correlation graphs for each group at each timepoint. As only the Miami cohort underwent exercise challenge GED comparisons were only made between groups within each time, and not across time within each group. Based on the GED measures indicated in Additional file 1: Table S7, the most similar graphs at rest were GWI_L_ and GWI_H_ with a GED measure of 107.40 while the most different were HC and GWI_L_ at 127.91. For peak exercise, the most similar graphs were HC and GWI_H_ with a GED measure of 116.93, while the most different were GWI_L_ and GWI_H_ at 118.88, closely followed by HC and GWI_L_ at 117.53. For post-exercise recovery, the GWI_L_ and GWI_H_ graphs were again the most similar with a GED measure of 86.07, with GWI_H_ and HC being the most different of 100.80. Overall, this suggests differences in the regulation of symptoms, hormones, and immune function between both GWI subgroups and with HC at all stages across an exercise challenge.

Specifically looking at the biological measures identified as showing significant differences between groups it was found that for HC at rest levels of NK % activity negatively correlated with SF36 role limitations due to physical problems (Spearman correlation coefficient ρ =  − 0.43, *P* = 0.0033, Storey *q* = 0.0382) while GWI_H_ showed negative correlation between IL-15 and MFI general fatigue (ρ =  − 0.42, *P* = 0.0025, *q* = 0.0253). For HC at peak exercise SF-36 role limitations due to physical problems negatively correlated with CD3^−^CD16^+^ (×10^9^/L) (ρ =  − 0.45, *P* = 0.0023, *q* = 0.0299), CD3^−^CD16^+^CD11a^+^ (%) (ρ =  − 0.43, *P* = 0.0037, q = 0.0425), and CD3^−^CD16^+^CD11a^+^ (×10^9^/L) (ρ =  − 0.46, *P* = 0.0015, *q* = 0.0224), while CD3^−^CD16^+^CD11a^+^ (%) also negatively correlated with SF-36 general health (ρ =  − 0.44, *P* = 0.0031, *q* = 0.0369). Finally, for GWI_L_ at peak exercise CD8^+^CD26^+^ cell percentage correlated negatively with MFI general fatigue (ρ =  − 0.73, *P* = 0.0041, *q* = 0.0150), reduced activity (ρ =  − 0.74, *P* = 0.0012, *q* = 0.0236) and reduced motivation (ρ =  − 0.77, *P* = 0.0003, *q* = 0.0090). No significant correlations were found between biological measures with significant groups differences and symptoms at the 4 h recovery point.

## Discussion

Research efforts to date concerning the link between GWI and PTSD have demonstrated that PTSD clinically appears to have clinical signs different from GWI [65], and that GWI comorbid with PTSD appears to exacerbate GWI symptomology [12, 13]. This research aimed to gain insight into the underlying pathophysiology of GWI and how comorbid PTSD impacts both symptom and biological presentation in this illness. This was achieved through stratifying GWI into trauma-based subgroups (GWI_L_ with low probability of PTSD, and GWI_H_ with high probability of comorbid PTSD) to determine subgroup specific symptom measures and biological markers such as hormones, cytokine, and immune cell measures. Our research efforts comparing symptom scales between GWI_L_ and GWI_H_ in an expanded cohort recapitulated our previous findings in males with GWI that GWI in the absence of PTSD (GWI_L_) presents with a significant set of symptoms compared to controls marking it as a distinct illness, which in the presence of PTSD (GWI_H_) presents with significantly worse symptoms overall [12]. Additionally, by comparing biological profiles we found that across GWI subgroups there were significantly lower BA levels at peak exercise making this a potential biomarker for GWI in general. GWI_L_ veterans were characterized distinctly from both GWI_H_ and HC groups by lower levels of IL-15 in general and reduced NK % activity and the related NK cell surface marker antigens CD3^−^CD56^+^, CD3^−^CD16^+^ and CD3^−^CD16^+^CD11a^+^ at peak exercise in addition to a higher CD2^+^CD26^+^ cell percentage at this same time. GWI_H_ veterans, on the other hand, were distinguished from controls by higher RDW, and a lower number of CD3^−^CD56^+^ and CD2^+^ cells and rest and lower HCT levels at peak exercise, but no significant differences from GWI_L_ other than for IL-15 at peak exercise. The low levels of IL-15 for GWI_L_ at all times compared to HC, and at peak exercise compared to GWI_H_ suggest it as a potential subgroup discriminating marker.

Here we found that BA % levels in GWI (0.15% for GWI_L_ and 0.19% for GWI_H_) were approximately half the level observed in controls at peak exercise (0.40%) (Additional file 3). While the determination of low BA levels is difficult, this reduced percentage in GWI is below the typical BA % in human peripheral blood of 0.5–1.0% [66], and outside the healthy reference range of 0.3–1.5% determined previously for the instrumentation used in this study. Research suggests that BA may also regulate T cells to mediate the magnitude of the secondary immune response [67], as well as regulate gut homeostasis [68]. The finding of low BA levels in GWI is consistent with a GWI altered immune profile [26], and more recent evidence suggests an altered gut-microbiome in GWI [69, 70] of which low BA levels may be a contributing factor. As this low level only occurs at peak exercise it may also be related to the exercise induced increase in symptoms and immune dysregulation observed in GWI [25, 29, 71–74]. Further investigation into this finding is needed to determine if low BA percentages at peak exercise are a consistent biomarker for GWI to distinguish from healthy sedentary Veteran controls.

The IL-15 levels of GWI_L_ veterans were found to be approximately 50—60% of HC, with GWI_H_ being comparable to the control level. This finding of GWI_L_ veterans having distinctly lower IL-15 levels from both GWI_H_ and HC groups is consistent with reduced NK levels at peak exercise (approximately 70% of control) as the cytokine IL-15 induces the proliferation of NK cells, and has been shown to increase following acute exercise [75–78], thus with lower levels of IL-15 to start, fewer NK cells would be produced. NK cells are innate lymphocytes that play important roles in the defense against microbial pathogens through recognition and lysis of virally or bacterially infected host cells, particularly the herpesvirus family [79], in locations such as the mucosal epithelia of the intestine, lung, and female reproductive tract [80]. Deficiency in these cells for the GWI_L_ group was only found to be significant at peak exercise. This suggests after exertion this group would be at increased susceptibility to new infection, or decreased control of an active or latent infection. This is similar to the GWI related illness myalgic encephalomyelitis/chronic fatigue syndrome (ME/CFS) for which herpesviruses have been implicated as possible etiological pathogens [81, 82], and for which there is evidence of reduced NK cell function [83–88] and IL-15 levels [48] correlating with increased illness severity [89–91], highlighting the similarity between these illnesses. Conversely, while NK cell number (CD3^−^CD56^+^ (×10^9^/L), and CD2^+^ (%) which includes both NK and T cells) was also observed in the GWI_H_ group at rest it was not accompanied by any significant changes in IL-15 at any time. One explanation for this difference between the GWI_H_ and GWI_L_ groups may be due to PTSD having been shown to have significantly elevated mean levels of IL-15 compared to controls [22]. This has been attributed to PTSD being associated with a genetic variant in the *il15* gene [92], and increased expression of the *il15* gene in the condition [93]. The apparent nominal level of IL-15 observed in GWI_H_ compared to control may be due to the combined, but opposite effects on IL-15, of GWI and PTSD when comorbid. Additionally, at peak exercise a approximately 15% increase in CD2^+^CD26^+^ cell percentage is observed for GWI_L_ compared to control. CD26 is a major contributor to the regulation of T, B, myeloid and NK cells and also plays a major role in T cell-dependent antibody production and immunoglobulin isotype switching in B cells, with abnormal expression of CD26 found in autoimmune diseases, rheumatologic, HIV-related illness and cancer [73, 94]. Normally NK cells usually express only low amounts of CD26 which increases after IL-15 stimulation [94]. Additionally, it appears that CD26 enzymatic activity sustains NK cytotoxicity [94]. An elevated percentage of CD2^+^CD26^+^ cells would suggest high levels of IL-15 and NK % activity; however, this is not what is observed here suggesting some dysregulation or dysfunction in this system in GWI_L_. Further study into IL-15 and NK cell counts and activity in GWI and PTSD are needed to determine if this is a possible subtyping biomarker with relevant biological implications.

The remaining difference between groups was found for GWI_H_ in RDW at rest and HCT at peak exercise compared to control. While both GWI_L_ and GWI_H_ veterans showed the same medium effect increase in RDW (g = 0.57, Additional file 1: Table S4) compared to HC, only the GWI_H_ reached the level of significance. Elevated levels of RDW indicate that there is a greater variation in the rheological properties of red blood cells such as size and volume. This is consistent with previous findings of abnormal rheological properties of red blood cells including higher shear stress, elongation, and elevation of RDW in GWI veterans [95]. An elevated RDW has been associated with fatigue in conditions such as systemic lupus erythematosus (SLE) [96] and post-stroke fatigue [97]. While the relation between RDW and fatigue is commonly related to iron deficiency and anemia [96, 98], like in SLE we found no significant changes in hemoglobin (HGB) that would support this. In addition to RDW elevation at rest, GWI_H_ was found to have lower hematocrit (HCT) levels at peak exercise. HCT is a measure of the % volume of red blood cells and is typically in the range of 39.5–50.3% for the instrumentation used in this study. While all groups had hematocrit values within this range the GWI_H_ condition had a statistically significantly higher level than control with a medium effect size. This lower level, however, is at odds with prior studies finding elevated HCT levels in PTSD compared to control [99–101]. While pathologically increased HCT is associated with hypercoagulability [102], and long-term risk of cardiovascular mortality [103], decreased HCT is associated with anemia. While these findings are contradictory, they highlight the importance of investigating circulating blood cell counts in GWI in the context of PTSD as potential illness markers. As nanoelectronics-blood-based diagnostics [104] are being used as a biomarker to detect red blood cell deformability in ME/CFS [105], in light of our findings similar methods may be applicable to GWI_._

Correlational analysis indicated differences between all groups in the coordinated regulation of cytokines, hormones, blood, and immune cells and how these relate to symptom presentation. This is consistent with previous network and graph-based analyses in GWI [25, 73, 106] showing differences in overall immune-symptom interactions. The results here show that this is extended further when GWI is subtyped based on PTSD symptoms and shows additional differences in immune-symptom interactions between trauma-based subgroups of GWI, as well as controls. While the significant correlations that were found between key biomarkers and symptoms were not found in all the groups, they did indicate that dysregulation of IL-15, NK % activity and CD3^−^CD16^+^ cells (with and without CD11a^+^) is related to role limitations due to physical problems, general health and general fatigue. As noted above there is an interplay between NK cells and IL-15 that is related to the symptoms observed in GWI and the related illness ME/CFS, in addition to noted genetic differences in IL-15 found in PTSD. As such further investigation is warranted into the effect of comorbid PTSD and GWI considering the direct influence of IL-15 and NK cell counts and activity on symptoms.

While the findings presented here support the need for subtyping of GWI based on PTSD symptoms, some limitations must be noted in both data collection and diagnosis classification for both GWI as well as PTSD. The main differences stem from the secondary analysis of a combination of two separate cohorts (Miami and Boston) recruited and assessed under different studies. The inclusion criteria in the two cohorts for both GWI and PTSD have some differences, especially as it relates to identification of subjects with PTSD (i.e. self-assessment versus psychiatric interview). As such, here we place our findings in the context of the groups having a high probability of having PTSD and PTSD symptoms, rather than a direct diagnosis. Future primary studies aimed at investigating the effects of PTSD on GWI should aim to use a clinical diagnosis of PTSD. Another limitation related to this is the lack of comparison to a PTSD cohort without GWI. Again, this stems directly from the secondary analysis nature of this study. Comparison against a PTSD cohort would further delineate symptoms and biomarkers that are unique to GWI, PTSD and comorbidities of these conditions.

Both maximal and submaximal exercise protocols have been used to determine behavioral and physiological consequences of acute exercise challenge in illnesses such as GWI, ME/CFS and long coronavirus disease [25, 71, 107–111]. Here we used exercise challenge to explore the consequences on GWI with and without PTSD symptomatology. It must be noted that only one of cohorts (i.e., the Miami cohort) underwent the exercise challenge. As such the sample size used for peak exercise and post-exercise measures is reduced compared to the at rest condition. However, while future primary study designs should aim to keep groups sizes consistent across the exercise challenge to allow for direct comparisons across time, our results indicate that group difference in IL-15 and CD3^−^CD56^+^ and CD3^−^CD16^+^ cells remain at different timepoints of the exercise challenge regardless of group size.

The final limitation of note concerns the cytokine results. Here we used cytokine values produced by the same Quansys system but using 16-plex and 18-plex panels. Due to the differences between these panels direct comparison of values is not appropriate and requires scaling of one data set before inclusion into the other. The result is a lack of accurate reporting of actual cytokine concentration levels in each group, however, the scaling procedure allows reliable statistical comparison of relative means between groups to identify potential biomarker differences.

## Conclusions

Based on the significance shown within our findings, this study supported that GWI with comorbid PTSD symptoms resulted in poorer health outcomes compared to both HC and the GWIL subgroup. Moreover, our findings further established that post-traumatic stress is not the driving factor behind the development and clinical presentation of GWI. Stratifying GWI veterans with PTSD and creating three cohorts HC, GWIL, and GWIH, enabled us to identify the relationship between each subgroup’s specific biological measures including hormone, immune cell and cytokine markers with the symptoms experienced. Significant differences were found in basophil levels of GWI compared to HC at peak exercise regardless of PTSD symptom comorbidity indicating its potential usage as a biomarker for general GWI from control. While the unique identification of GWI with PTSD symptoms was less clear, the GWIL subgroup was found to be delineated from both GWIH and control on measures of IL-15 across an exercise challenge. This, the additional differences in NK cell numbers and function and the genetic risk factor of variants of IL-15 highlight this cytokine as a potential biomarker of GWI in the absence of PTSD symptoms. This in turn may provide necessary insight and direction for symptom management and treatment for each distinct group.

### Supplementary Information

**Additional file 1**. **Table S1** Antibody cocktails and fluorochrome used for Flow cytometry. **Table S2** Cohort demographics. **Table S3** Welch ANOVA Omnibus significant comparisons of symptom scales rest between trauma defined GWI groups and controls for the combined Miami and Boston cohorts. **Table S4** Welch ANOVA Omnibus significant comparisons of hormones, cytokines, complete blood count and flow cytometry measures at rest between trauma defined GWI groups and controls for the combined Miami and Boston cohorts. **Table S5** Welch ANOVA Omnibus significant comparisons of hormones, cytokines, complete blood count and flow cytometry measures at peak exercise between trauma defined GWI groups and controls for the Miami cohort. **Table S6** Welch ANOVA Omnibus significant comparisons of hormones, cytokines, complete blood count and flow cytometry measures at 4 h post exercise between trauma defined GWI groups and controls for the Miami cohort. **Table S7** Graph edit distance (GED) comparison between GWI subgroups and controls across time. **Fig. S1** Correlation graph for HC at rest. Spearman correlation ρ values range between -1 and 1 as indicated in the colormap on the right. **Fig. S2** Correlation graph for GWIL at rest. **Fig. S3** Correlation graph for GWIH at rest. **Fig. S4** Correlation graph for HC at peak exercise. **Fig. S5** Correlation graph for GWIL at peak exercise. **Fig. S6** Correlation graph for GWIH at peak exercise. **Fig. S7** Correlation graph for HC 4 h post exercise. **Fig. S8** Correlation graph for GWIL 4 h post exercise. **Fig. S9** Correlation graph for GWIH 4 h post exercise.

**Additional file 2**. Flow cytometry gating strategy.

**Additional file 3**. Complete data and analysis.

## Data Availability

The data that support the findings of this study are from the MVAMC and the Boston biorepository, recruitment and integrative network (BBRAIN) for GWI at Boston University, but restrictions apply to the availability of these data, which were used under license for the current study, and so are not publicly available. Data are however available from the authors upon reasonable request and with permission of the MVAMC and the BBRAIN.

## Abbreviations

a.u.: Arbitrary units
ANOVA: Analysis of variance
BA: Basophils
BMI: Body mass index
CAPS-5: Clinically Administered PTSD Scale for DSM-5
CBC: Complete blood count
CD: Cluster of differentiation
DoD: Department of Defense
DSM: Diagnostic and statistical manual of mental disorders
DTS: Davidson Trauma Scale
EDTA: Ethylenediaminetetraacetic acid
ELISA: Enzyme-linked immunosorbent assay
GED: Graph edit distance
GWI: Gulf War Illness
GWIC: Gulf War Illness Consortium
GWI_H_: Gulf War Illness with high probability of PTSD symptoms
GWI_L_: Gulf War Illness with low probability of PTSD symptoms
HC: Healthy control
HCT: Hematocrit
HGB: Hemoglobin
HRPO: Human Research Protections Office
IFN: Interferon
IL: Interleukin
IRB: Institutional review board
ME/CFS: Myalgic encephalomyelitis/Chronic fatigue syndrome
MFI: Multidimensional fatigue inventory
MVAMC: Miami Veterans Affairs Medical Center
NK: Natural killer
NSU: Nova Southeastern University
PTSD: Post-traumatic stress disorder
RDW: Red blood cell distribution width
SCID: Structured Clinical Interview for DSM-5
SF-36: 36-Item short form health survey
T0: Timepoint 0 at rest
T1: Timepoint 1 at peak exercise
T2: Timepoint 2–4 h after peak exercise
TNF: Tumor necrosis factor
USAMRMC: United States Army Medical Research and Material Command

## References

[1] Binns J, Barlow C, Bloom F, Clauw D, Golomb B, Graves J, et al. Research advisory committee on Gulf War veterans' illnesses. Gulf War Illness and the health of gulf War veterans. Washington, DC: Department of Veterans Affairs. 2008.

[2] Dickey B, Madhu LN, Shetty AK (2020). Gulf War Illness: mechanisms underlying brain dysfunction and promising therapeutic strategies. Pharmacol Ther.

[3] Nicolson GL (2001). Gulf War Illnesses: causes and treatments. Armed Forces Med Dev.

[4] Fujita M, Southwick SM, Denucci CC, Zoghbi SS, Dillon MS, Baldwin RM (2004). Central type benzodiazepine receptors in Gulf War veterans with posttraumatic stress disorder. Biol Psychiatry.

[5] Boscarino JA (2008). A prospective study of PTSD and early-age heart disease mortality among Vietnam veterans: implications for surveillance and prevention. Psychosom Med.

[6] American Psychiatric Association (2013). DSM-5 Task Force. Diagnostic and statistical manual of mental disorders: DSM-5™.

[7] Pacella ML, Hruska B, Delahanty DL (2013). The physical health consequences of PTSD and PTSD symptoms: a meta-analytic review. J Anxiety Disord.

[8] Xenakis SN (2014). Posttraumatic stress disorder: Beyond best practices. Psychoanal Psychol.

[9] Hoge CW, Terhakopian A, Castro CA, Messer SC, Engel CC (2007). Association of posttraumatic stress disorder with somatic symptoms, health care visits, and absenteeism among Iraq war veterans. Am J Psychiatry.

[10] Rischardson L, Frueh B, Acierno R (2010). Prevalence estimates of combat-related post-traumatic stress disorder: critical review. Aust N Z J Psychiatry.

[11] Weiner MW, Meyerhoff DJ, Neylan TC, Hlavin J, Ramage ER, McCoy D (2011). The relationship between Gulf War Illness, brain N-acetylaspartate, and post-traumatic stress disorder. Mil Med.

[12] Jeffrey M, Collado F, Kibler J, DeLucia C, Messer S, Klimas N (2021). Post-traumatic stress impact on health outcomes in Gulf War Illness. BMC Psychol.

[13] Shastry N, Sultana E, Jeffrey M, Collado F, Kibler J, DeLucia C (2022). The impact of post-traumatic stress on symptom presentation of women with Gulf War Illness. BMC Psychol.

[14] Moeller-Bertram T, Keltner J, Strigo IA (2012). Pain and post traumatic stress disorder–review of clinical and experimental evidence. Neuropharmacology.

[15] Agorastos A, Boel JA, Heppner PS, Hager T, Moeller-Bertram T, Haji U (2013). Diminished vagal activity and blunted diurnal variation of heart rate dynamics in posttraumatic stress disorder. Stress.

[16] Moeller-Bertram T, Afari N, Mostoufi S, Fink DS, Wright LJ, Baker DG (2014). Specific pain complaints in Iraq and Afghanistan veterans screening positive for post-traumatic stress disorder. Psychosomatics.

[17] Paulus EJ, Argo TR, Egge JA (2013). The impact of posttraumatic stress disorder on blood pressure and heart rate in a veteran population. J Trauma Stress.

[18] Plantinga L, Bremner JD, Miller AH, Jones DP, Veledar E, Goldberg J (2013). Association between posttraumatic stress disorder and inflammation: a twin study. Brain Behav Immun.

[19] Baker DG, Nievergelt CM, O'Connor DT (2012). Biomarkers of PTSD: neuropeptides and immune signaling. Neuropharmacology.

[20] Pace TW, Heim CM (2011). A short review on the psychoneuroimmunology of posttraumatic stress disorder: from risk factors to medical comorbidities. Brain Behav Immun.

[21] Guo M, Liu T, Guo J-C, Jiang X-L, Chen F, Gao YS (2012). Study on serum cytokine levels in posttraumatic stress disorder patients. Asian Pac J Trop Med.

[22] Hoge EA, Brandstetter K, Moshier S, Pollack MH, Wong KK, Simon NM (2009). Broad spectrum of cytokine abnormalities in panic disorder and posttraumatic stress disorder. Depress Anxiety.

[23] Zhang L, Hu XZ, Li X, Chen Z, Benedek DM, Fullerton CS (2020). Potential chemokine biomarkers associated with PTSD onset, risk and resilience as well as stress responses in US military service members. Transl Psychiatry.

[24] Nkiliza A, Joshi U, Evans JE, Ait-Ghezala G, Parks M, Crawford F (2021). Adaptive immune responses associated with the central nervous system pathology of Gulf War Illness. Neurosci Insights.

[25] Broderick G, Ben-Hamo R, Vashishtha S, Efroni S, Nathanson L, Barnes Z (2013). Altered immune pathway activity under exercise challenge in Gulf War Illness: an exploratory analysis. Brain Behav Immun.

[26] Steele L, Klimas N, Krengel M, Quinn E, Toomey R, Little D (2021). Brain–immune interactions as the basis of Gulf War Illness: Clinical assessment and deployment profile of 1990–1991 Gulf War veterans in the Gulf War Illness consortium (GWIC) multisite case-control study. Brain Sci.

[27] Smylie AL, Broderick G, Fernandes H, Razdan S, Barnes Z, Collado F (2013). A comparison of sex-specific immune signatures in Gulf War Illness and chronic fatigue syndrome. BMC Immunol.

[28] Parkitny L, Middleton S, Baker K, Younger J (2015). Evidence for abnormal cytokine expression in Gulf War Illness: A preliminary analysis of daily immune monitoring data. BMC Immunol.

[29] Whistler T, Fletcher MA, Lonergan W, Zeng X-R, Lin J-M, LaPerriere A (2009). Impaired immune function in Gulf War Illness. BMC Med Genomics.

[30] Haley RW, Kramer G, Xiao J, Dever JA, Teiber JF (2022). Evaluation of a gene–environment interaction of *PON1* and low-level nerve agent exposure with Gulf War Illness: a prevalence case–control study drawn from the US Military Health Survey’s national population sample. Environ Health Perspect.

[31] Vahey J, Gifford EJ, Sims KJ, Chesnut B, Boyle SH, Stafford C (2021). Gene–toxicant interactions in Gulf War Illness: differential effects of the *PON1* genotype. Brain Sci.

[32] Haines DD, Ottenweller JE, Dickens BF, Mahmoud FF, Levine PH (2017). Activity of paraoxonase/arylesterase and butyrylcholinesterase in peripheral blood of Gulf War era veterans with neurologic symptom complexes or PTSD. J Occup Environ Med.

[33] Moreira EG, Boll KM, Correia DG, Soares JF, Rigobello C, Maes M (2019). Why should psychiatrists and neuroscientists worry about paraoxonase 1?. Curr Neuropharmacol.

[34] Ogłodek EA (2017). The role of PON-1, GR, IL-18, and OxLDL in depression with and without posttraumatic stress disorder. Pharmacol Rep.

[35] Atli A, Bulut M, Bez Y, Kaplan I, Özdemir PG, Uysal C (2016). Altered lipid peroxidation markers are related to post-traumatic stress disorder (PTSD) and not trauma itself in earthquake survivors. Eur Arch Psychiatry Clin Neurosci.

[36] Fukuda K, Nisenbaum R, Stewart G, Thompson WW, Robin L, Washko RM (1998). Chronic multisymptom illness affecting Air Force veterans of the Gulf War. JAMA.

[37] Reeves WC, Lloyd A, Vernon SD, Klimas N, Jason LA, Bleijenberg G (2003). Identification of ambiguities in the 1994 chronic fatigue syndrome research case definition and recommendations for resolution. BMC Health Serv Res.

[38] Collins JF, Donta ST, Engel CC, Baseman JB, Dever LL, Taylor T (2002). The antibiotic treatment trial of Gulf War Veterans' Illnesses: issues, design, screening, and baseline characteristics. Controlled Clin Trials.

[39] Keating D, Zundel C, Abreu M, Krengel M, Aenlle K, Nichols M (2021). Boston biorepository, recruitment and integrative network (BBRAIN): a resource for the Gulf War Illness scientific community. Life Sci.

[40] Steele L (2000). Prevalence and patterns of Gulf War Illness in Kansas veterans: association of symptoms with characteristics of person, place, and time of military service. Am J Epidemiol.

[41] Davidson JR, Book S, Colket J, Tupler L, Roth S, David D (1997). Assessment of a new self-rating scale for post-traumatic stress disorder. Psychol Med.

[42] McDonald SD, Thompson NL, Stratton KJ, Calhoun PS (2014). Diagnostic accuracy of three scoring methods for the Davidson Trauma Scale among US military Veterans. J Anxiety Disord.

[43] Weathers FW, Bovin MJ, Lee DJ, Sloan DM, Schnurr PP, Kaloupek DG (2018). The Clinician-Administered PTSD Scale for DSM–5 (CAPS-5): Development and initial psychometric evaluation in military veterans. Psychol Asses.

[44] Williams M, Karg R, Spitzer R (2015). Structured clinical interview for DSM-5–research version (SCID-5 for DSM-5, research version; SCID-5–RV).

[45] Smets E, Garssen B, Bonke Bd, De Haes J (1995). The Multidimensional Fatigue Inventory (MFI) psychometric qualities of an instrument to assess fatigue. J Psychosom Res.

[46] Ware J, Sherbourne C (1992). The MOS 36-item short-form health survey (SF-36) I: conceptual framework and item selection. Med Care.

[47] McHorney CA, Ware JE, Raczek AE (1993). The MOS 36-Item Short-Form Health Survey (SF-36): II Psychometric and clinical tests of validity in measuring physical and mental health constructs. Med Care.

[48] Fletcher MA, Zeng XR, Barnes Z, Levis S, Klimas NG (2009). Plasma cytokines in women with chronic fatigue syndrome. J Transl Med.

[49] Maher K, Klimas N, Fletcher M (2003). Immunology of chronic fatigue syndrome, Handbook of chronic fatigue syndrome.

[50] Fletcher MA, Zeng XR, Maher K, Levis S, Hurwitz B, Antoni M (2010). Biomarkers in chronic fatigue syndrome: evaluation of natural killer cell function and dipeptidyl peptidase IV/CD26. PLoS One.

[51] Welch BL (1951). On the comparison of several mean values: an alternative approach. Biometrika.

[52] Tomarken AJ, Serlin RC (1986). Comparison of ANOVA alternatives under variance heterogeneity and specific noncentrality structures. Psychol Bull.

[53] Penn A. wanova. In: MATLAB central file exchange (https://www.mathworks.com/matlabcentral/fileexchange/61661-wanova). Accessed on July 12, 2023.

[54] Nagy P. Cumulative distribution function of the studentized range (for Tukey's HSD test). In: MATLAB Central File Exchange. (https://www.mathworks.com/matlabcentral/fileexchange/37450-cumulative-distribution-function-of-the-studentized-range-for-tukey-s-hsd-test). Accessed on July 12, 2023.

[55] Mégevand P. pierremegevand/games_howell. In: GitHub (https://github.com/pierremegevand/games_howell). Accessed July 12, 2023.

[56] Lund R, Lund J (1983). Algorithm AS 190: probabilities and upper quantiles for the studentized range. J R Stat Soc Ser C Appl Stat.

[57] Hedges LV (1981). Distribution theory for Glass's estimator of effect size and related estimators. J Educ Stat.

[58] Sawilowsky SS (2009). New effect size rules of thumb. J Mod Appl Stat Methods.

[59] Sullivan GM, Feinn R (2012). Using effect size—or why the *P* value is not enough. J Grad Med Educ.

[60] Storey JD (2002). A direct approach to false discovery rates. J R Stat Soc Series B Stat Methodol.

[61] Serratosa F (2019). Graph edit distance: Restrictions to be a metric. Pattern Recognit.

[62] Serratosa F (2021). Redefining the graph edit distance. SN Comput Sci.

[63] Bunke H (1983). What is the distance between graphs. Bull EATCS.

[64] Bunke H. Graph matching: Theoretical foundations, algorithms, and applications. In: Proc Vision Interface; 2000. p.82–8.

[65] Kang HK, Mahan CM, Lee KY, Murphy FM, Simmens SJ, Young HA (2002). Evidence for a deployment-related Gulf War syndrome by factor analysis. Arch Environ Health.

[66] Shah H, Eisenbarth S, Tormey CA, Siddon AJ (2021). Behind the scenes with basophils: an emerging therapeutic target. Immunother Adv..

[67] Nakanishi K (2010). Basophils as APC in Th2 response in allergic inflammation and parasite infection. Curr Opin Immunol.

[68] Bandaletova T, Chhaya V, Poullis A, Loktionov A (2016). Colorectal mucus non-invasively collected from patients with inflammatory bowel disease and its suitability for diagnostic cytology. APMIS.

[69] Janulewicz PA, Seth RK, Carlson JM, Ajama J, Quinn E, Heeren T (2019). The gut-microbiome in Gulf War veterans: a preliminary report. Int J Environ Res Public Health.

[70] Slevin E, Koyama S, Harrison K, Wan Y, Klaunig JE, Wu C (2023). Dysbiosis in gastrointestinal pathophysiology: Role of the gut microbiome in Gulf War Illness. J Cell Mol Med.

[71] Boruch AE, Lindheimer JB, Klein-Adams JC, Stegner AJ, Wylie GR, Ninneman JV (2021). Predicting post-exertional malaise in Gulf War Illness based on acute exercise responses. Life Sci.

[72] Lindheimer JB, Stegner AJ, Wylie GR, Klein-Adams JC, Almassi NE, Ninneman JV (2020). Post-exertional malaise in veterans with gulf war illness. Int J Psychophys.

[73] Broderick G, Kreitz A, Fuite J, Fletcher MA, Vernon SD, Klimas N (2011). A pilot study of immune network remodeling under challenge in Gulf War Illness. Brain Behav Immun.

[74] Arias FJC, Aenlle K, Abreu M, Holschbach MA, Michalovicz LT, Kelly KA (2021). Modeling Neuroimmune interactions in human subjects and animal models to predict subtype-specific multidrug treatments for Gulf War Illness. Int J Mol Sci.

[75] Riechman SE, Balasekaran G, Roth SM, Ferrell RE (2004). Association of interleukin-15 protein and interleukin-15 receptor genetic variation with resistance exercise training responses. J Appl Physiol.

[76] Tamura Y, Watanabe K, Kantani T, Hayashi J, Ishida N, Kaneki M (2011). Upregulation of circulating IL-15 by treadmill running in healthy individuals: is IL-15 an endocrine mediator of the beneficial effects of endurance exercise?. Endocr J.

[77] Crane JD, MacNeil LG, Lally JS, Ford RJ, Bujak AL, Brar IK (2015). Exercise-stimulated interleukin-15 is controlled by AMPK and regulates skin metabolism and aging. Aging Cell.

[78] Luo Z, He Z, Qin H, Chen Y, Qi B, Lin J (2022). Exercise-induced IL-15 acted as a positive prognostic implication and tumor-suppressed role in pan-cancer. Front Pharmacol.

[79] Orange JS (2013). Natural killer cell deficiency. J Allergy Clin Immunol.

[80] Fuchs A, Colonna M (2011). Natural killer (NK) and NK-like cells at mucosal epithelia: mediators of anti-microbial defense and maintenance of tissue integrity. Eur J Microbiol Immunol.

[81] Ariza ME (2021). Myalgic encephalomyelitis/chronic fatigue syndrome: the human herpesviruses are back!. Biomolecules.

[82] Cox BS, Alharshawi K, Mena-Palomo I, Lafuse WP, Ariza ME (2022). EBV/HHV-6A dUTPases contribute to myalgic encephalomyelitis/chronic fatigue syndrome pathophysiology by enhancing TFH cell differentiation and extrafollicular activities. JCI Insight.

[83] Barker E, Fujimura SF, Fadem MB, Landay AL, Levy JA (1994). Immunologic abnormalities associated with chronic fatigue syndrome. Clin Infect Dis.

[84] Whiteside TL, Friberg D (1998). Natural killer cells and natural killer cell activity in chronic fatigue syndrome. Am J Med.

[85] Fletcher MA, Maher KJ, Klimas NG (2002). Natural killer cell function in chronic fatigue syndrome. Clin Appl Immunol Rev.

[86] Brenu EW, Van Driel ML, Staines DR, Ashton KJ, Hardcastle SL, Keane J (2012). Longitudinal investigation of natural killer cells and cytokines in chronic fatigue syndrome/myalgic encephalomyelitis. J Transl Med.

[87] Brenu EW, Huth TK, Hardcastle SL, Fuller K, Kaur M, Johnston S (2014). Role of adaptive and innate immune cells in chronic fatigue syndrome/myalgic encephalomyelitis. Int Immunol.

[88] Klimas NG, Salvato FR, Morgan R, Fletcher MA (1990). Immunologic abnormalities in chronic fatigue syndrome. J Clin Microbiol.

[89] Ojo-Amaize EA, Conley EJ, Peter JB (1994). Decreased natural killer cell activity is associated with severity of chronic fatigue immune dysfunction syndrome. Clin Infect Dis.

[90] Strayer D, Scott V, Carter W (2015). Low NK cell activity in chronic fatigue syndrome (CFS) and relationship to symptom severity. J Clin Cell Immunol.

[91] Hornig M, Montoya JG, Klimas NG, Levine S, Felsenstein D, Bateman L (2015). Distinct plasma immune signatures in ME/CFS are present early in the course of illness. Sci Adv.

[92] Wang Y, Karstoft K-I, Nievergelt CM, Maihofer AX, Stein MB, Ursano RJ (2019). Post-traumatic stress following military deployment: Genetic associations and cross-disorder genetic correlations. J Affect Disord.

[93] Chitrala KN, Nagarkatti P, Nagarkatti M (2016). Prediction of possible biomarkers and novel pathways conferring risk to post-traumatic stress disorder. PLoS One.

[94] Klemann C, Wagner L, Stephan M, von Hörsten S (2016). Cut to the chase: a review of CD26/dipeptidyl peptidase-4's (DPP4) entanglement in the immune system. Clin Exp Immunol.

[95] Falvo MJ, Chen Y, Klein JC, Ndirangu D, Condon MR (2018). Abnormal rheological properties of red blood cells as a potential marker of Gulf War Illness: a preliminary study. Clin Hemorheol Microcirc.

[96] Wincup C, Parnell C, Cleanthous S, Tejera Segura B, Nguyen MH, Bryant K (2019). Red cell distribution width correlates with fatigue levels in a diverse group of patients with systemic lupus erythematosus irrespective of anaemia status. Clin Exp Rheumatol.

[97] Peng M, Chen Y, Chen Y, Feng K, Shen H, Huang H (2022). The relationship between red blood cell distribution width at admission and post-stroke fatigue in the acute phase of acute ischemic stroke. Front Neurol.

[98] Badrick T, Richardson AM, Arnott A, Lidbury BA (2015). The early detection of anaemia and aetiology prediction through the modelling of red cell distribution width (RDW) in cross-sectional community patient data. Diagnosis.

[99] Lindqvist D, Mellon SH, Dhabhar FS, Yehuda R, Grenon SM, Flory JD (2017). Increased circulating blood cell counts in combat-related PTSD: Associations with inflammation and PTSD severity. Psychiatry Res.

[100] Mellon SH, Bersani FS, Lindqvist D, Hammamieh R, Donohue D, Dean K, J, (2019). Metabolomic analysis of male combat veterans with post traumatic stress disorder. PLoS One.

[101] Miranda O, Fan P, Qi X, Yu Z, Ying J, Wang H (2022). DeepBiomarker: identifying important lab tests from electronic medical records for the prediction of suicide-related events among PTSD patients. J Pers Med.

[102] Schreijer AJ, Reitsma PH, Cannegieter SC (2010). High hematocrit as a risk factor for venous thrombosis Cause or innocent bystander?. Haematologica.

[103] Skretteberg PT, Bodegård J, Kjeldsen SE, Erikssen G, Thaulow E, Sandvik L (2010). Interaction between inflammation and blood viscosity predicts cardiovascular mortality. Scand Cardiovasc J.

[104] Esfandyarpour R, Kashi A, Nemat-Gorgani M, Wilhelmy J, Davis R (2019). A nanoelectronics-blood-based diagnostic biomarker for myalgic encephalomyelitis/chronic fatigue syndrome (ME/CFS). Proc Natl Acad Sci.

[105] Saha AK, Schmidt BR, Wilhelmy J, Nguyen V, Abugherir A, Do JK (2019). Red blood cell deformability is diminished in patients with Chronic Fatigue Syndrome. Clin Hemorheol Microcirc.

[106] Broderick G, Fletcher MA, Gallagher M, Barnes Z, Vernon SD, Klimas NG (2012). Exploring the diagnostic potential of immune biomarker coexpression in Gulf War Illness.

[107] Joseph P, Singh I, Oliveira R, Capone CA, Mullen MP, Cook DB (2023). Exercise pathophysiology in myalgic encephalomyelitis/chronic fatigue syndrome and post-acute sequelae of SARS-CoV-2: more in common than not?. Chest.

[108] Peterson PK, Sirr S, Grammith F, Schenck C, Pheley A, Hu S (1994). Effects of mild exercise on cytokines and cerebral blood flow in chronic fatigue syndrome patients. Clin Diagn Lab Immunol.

[109] Rayhan RU, Stevens BW, Raksit MP, Ripple JA, Timbol CR, Adewuyi O (2013). Exercise challenge in Gulf War Illness reveals two subgroups with altered brain structure and function. PLoS One.

[110] Lyman CA, Clement M, Craddock TJ, Fletcher M, Klimas NG, Broderick G, editors. Feedback regulation of immune response to maximum exercise in Gulf War Illness. Proceedings of the 10th ACM International conference on bioinformatics, computational biology and health informatics; 2019: ACM.

[111] Friedberg F, Adamowicz JL, Bruckenthal P, Milazzo M, Ramjan S, Zhang X (2023). Sex differences in post-exercise fatigue and function in myalgic encephalomyelitis/chronic fatigue syndrome. Sci Rep.

