# Supplementary material for: Disentangling the effects of PTSD from Gulf War Illness in male veterans via a systems-wide analysis of immune cell, cytokine, and symptom measures
Source: Mil Med Res. 2024 Jan 2;11:2. doi: 10.1186/s40779-023-00505-4 (PMC10759613; doi:10.1186/s40779-023-00505-4)
Supplement: Supplementary file 1 — Additional file 1. Table S1 Antibody cocktails and fluorochrome used for Flow cytometry. Table S2 Cohort demographics. Table S3 Welch ANOVA Omnibus significant comparisons of symptom scales rest between trauma defined GWI groups and controls for the combined Miami and Boston cohorts. Table S4 Welch ANOVA Omnibus significant comparisons of hormones, cytokines, complete blood count and flow cytometry measures at rest between trauma defined GWI groups and controls for the combined Miami and Boston cohorts. Table S5 Welch ANOVA Omnibus significant comparisons of hormones, cytokines, complete blood count and flow cytometry measures at peak exercise between trauma defined GWI groups and controls for the Miami cohort. Table S6 Welch ANOVA Omnibus significant comparisons of hormones, cytokines, complete blood count and flow cytometry measures at 4 h post exercise between trauma defined GWI groups and controls for the Miami cohort. Table S7 Graph edit distance (GED) comparison between GWI subgroups and controls across time. Fig. S1 Correlation graph for HC at rest. Spearman correlation ρ values range between -1 and 1 as indicated in the colormap on the right. Fig. S2 Correlation graph for GWIL at rest. Fig. S3 Correlation graph for GWIH at rest. Fig. S4 Correlation graph for HC at peak exercise. Fig. S5 Correlation graph for GWIL at peak exercise. Fig. S6 Correlation graph for GWIH at peak exercise. Fig. S7 Correlation graph for HC 4 h post exercise. Fig. S8 Correlation graph for GWIL 4 h post exercise. Fig. S9 Correlation graph for GWIH 4 h post exercise. [file 40779_2023_505_MOESM1_ESM.pdf]

Supporting Information: Tables S1-S7 and Fig. S1-S9

Table S1 Antibody cocktails and fluorochrome used for flow cytometry

Antibody cocktails	Name of antibodies/isotype controls/target antigens	Fluorochromes
1	CD45	FITC
	CD4	RD1/PE
	CD19	ECD
	CD14	PC5
2	IgG1 mouse isotype control	FITC
	IgG1 mouse isotype control	RD1/PE
	IgG1 mouse isotype control	ECD
	IgG1 mouse isotype control	PC5
3	CD3	FITC
	CD56	RD1/PE
	CD4	ECD
	CD8	PC5
4	CD2	FITC
	CD26	RD1/PE
	CD4	ECD
	CD8	PC5
5	CD16	FITC
	CD11a	RD1/PE
	CD8	ECD
	CD3	PC5

CD cluster of differentiation, *ECD* phycoerythrin-Texas Red-X dye, *FITC* fluorescein isothiocyanate, *Ig* immunoglobulin, *PC5* phycoerythrin-cyanin 5.1, *RD1/PE* phycoerythrin

Table S2 Cohort demographics

Item	Total (n=120)	GWI (n=76)	GWI_H (n=47)	GWI_L (n=29)	HC (n=44)	P_3	P_2
Mean age (year, M ± SD)	46.0 ± 7.0	46.8 ± 7.4	46.4 ± 5.4	47.4 ± 9.8	44.5 ± 6.1	0.180	0.080
Mean BMI (kg/m^2, M ± SD)	30.29 ± 4.80	30.84 ± 5.04	30.61 ± 4.76	31.22 ± 5.54	29.19 ± 4.11	0.163	0.068
Race [n (%)]						0.136	0.036*
Asian	2(1.7)	2(2.6)	1(2.1)	1(3.4)	0(0.0)		
Black	24(20.0)	17(22.4)	12(25.5)	5(17.2)	7(15.9)		
White Hispanic	49(40.8)	23(30.3)	14(29.8)	9(31.0)	26(59.1)		
White	41(34.2)	31(40.8)	19(40.4)	12(41.4)	10(22.7)		
Other	4(3.3)	3(3.9)	1(2.1)	2(6.9)	1(2.3)		
Mean Schooling (year, M ± SD)	14.08 ± 4.83	14.27 ± 4.14	15.02 ± 2.33	13.00 ± 5.93	13.71 ± 6.01	0.119	0.585

GWI Gulf War Illness, GWI_H Gulf War Illness with high probability of PTSD symptoms, GWI_L Gulf War Illness with low probability of PTSD symptoms, *HC* healthy control, P_2 statistical test between GWI and HC, P_3 statistical test between GWI_H, GWI_L and HC. $^*P < 0.05$

Table S3 Welch ANOVA Omnibus significant comparisons of symptom scales rest between trauma defined GWI groups and controls for the combined Miami and Boston cohorts

Measure	Mean ± SEM			*P*-value			g		
	HC	GWI_L	GWI_H	HC/GWI_L	HC/GWI_H	GWI_L/GWI_H	HC/GWI_L	HC/GWI_H	GWI_L/GWI_H
MFI									
General fatigue	18.09 ± 2.53	60.42 ± 4.71	78.53 ± 2.73	$1.08\times10^{-3*}$	$6.59\times10^{-4*}$	$5.69\times10^{-3*}$	2.03	3.37	0.83
Physical fatigue	13.95 ± 2.56	53.35 ± 6.08	69.50 ± 3.11	$1.14\times10^{-3*}$	$6.60\times10^{-4*}$	$6.34\times10^{-2*}$	1.60	2.84	0.61
Mental fatigue	16.47 ± 3.29	58.48 ± 4.69	76.55 ± 2.94	$1.01\times10^{-3*}$	$6.62\times10^{-4*}$	$1.52\times10^{-2*}$	1.66	2.84	0.75
Reduced activity	15.12 ± 2.18	50.85 ± 4.91	62.09 ± 3.17	$1.14\times10^{-3*}$	$6.80\times10^{-4*}$	$1.42\times10^{-*}$	1.83	2.50	0.48
Reduced motivation	12.79 ± 2.07	44.12 ± 5.43	61.46 ± 3.46	$1.14\times10^{-3*}$	$7.21\times10^{-4*}$	$1.80\times10^{-2*}$	1.57	2.47	0.69
SF-36									
Physical function	90.83 ± 2.25	63.21 ± 7.28	48.00 ± 4.01	$1.27\times10^{-3*}$	$8.45\times10^{-4*}$	7.87×10^{-2}	1.26	1.90	0.53
Role physical	90.34 ± 3.74	43.89 ± 4.48	22.42 ± 4.12	$1.11\times10^{-3*}$	$6.62\times10^{-4*}$	$3.70\times10^{-2*}$	1.47	2.53	0.65
Pain	81.42 ± 3.01	42.68 ± 3.85	32.93 ± 3.27	$9.82\times10^{-4*}$	$6.60\times10^{-4*}$	2.04×10^{-1}	1.77	2.26	0.42
General health	78.86 ± 2.51	39.31 ± 4.45	29.15 ± 2.93	$9.82\times10^{-4*}$	$6.59\times10^{-4*}$	9.91×10^{-2}	2.13	2.67	0.50
Vitality	70.36 ± 2.55	33.75 ± 5.90	20.68 ± 2.36	$1.06\times10^{-3*}$	$6.65\times10^{-4*}$	$3.92\times10^{-2*}$	1.81	2.97	0.66
Social functioning	90.91 ± 2.20	57.59 ± 7.79	32.61 ± 3.76	$1.22\times10^{-3*}$	$7.34\times10^{-4*}$	$3.61\times10^{-3*}$	1.44	2.75	0.88
Role emotional	96.21 ± 1.94	57.74 ± 3.08	27.36 ± 4.97	$1.44\times10^{-3*}$	$9.11\times10^{-4*}$	$6.83\times10^{-3*}$	1.36	2.63	0.81
Mental health	84.55 ± 1.76	60.00 ± 1.59	42.47 ± 2.94	$1.14\times10^{-3*}$	$7.19\times10^{-4*}$	$1.27\times10^{-3*}$	1.77	2.52	0.92

g corrected hedge's g effect size, *GWI* Gulf War Illness, GWI_H Gulf War Illness with high probability of PTSD symptoms, GWI_L Gulf War Illness with low probability of PTSD symptoms, *HC* healthy control, *P* pairwise significance measure as determined by a Games-Howell post-hoc analysis, *SEM* standard error of the mean. $^*P < 0.05$

Table S4 Welch ANOVA Omnibus significant comparisons of hormones, cytokines, complete blood count and flow cytometry measures at rest between trauma defined GWI groups and controls for the combined Miami and Boston cohorts

Measure (units)	Mean ± SEM			*P*-value			g		
	HC	GWI_L	GWI_H	HC/GWI_L	HC/GWI_H	GWI_L /GWI_H	HC/GWI_L	HC/GWI_H	GWI_L /GWI_H
hIL-1b (a.u.)	0.14 ± 0.02	0.08 ± 0.01	0.13 ± 0.03	5.699×10^{-2}	9.50×10^{-1}	2.63×10^{-1}	0.49	0.06	0.31
hIL-15 (a.u.)	0.24 ± 0.03	0.15 ± 0.02	0.22 ± 0.03	$2.63\times10^{-2*}$	8.96×10^{-1}	1.21×10^{-1}	0.58	0.09	0.40
RDW (%)	12.74 ± 0.12	13.33 ± 0.24	13.29 ± 0.16	8.37×10^{-2}	$1.88\times10^{-2*}$	9.90×10^{-1}	0.57	0.57	0.03
CD3-CD56+# ($\times10^9$ /L)	201.48 ± 17.94	161.24 ± 12.47	148.00 ± 11.44	1.64×10^{-1}	$3.85\times10^{-2*}$	7.18×10^{-1}	0.39	0.53	0.18
$CD2^+$ (%)	82.76 ± 0.71	78.05 ± 1.82	78.54 ± 1.10	5.49×10^{-2}	$6.46\times10^{-3*}$	9.71×10^{-1}	0.65	0.66	0.06

g corrected hedge's g effect size, *GWI* Gulf War Illness, GWI_H Gulf War Illness with high probability of PTSD symptoms, GWI_L Gulf War Illness with low probability of PTSD symptoms, *HC* healthy control, *P* pairwise significance measure as determined by a Games-Howell post-hoc analysis, *SEM* standard error of the mean. $^*P < 0.05$

Table S5 Welch ANOVA Omnibus significant comparisons of hormones, cytokines, complete blood count and flow cytometry measures at peak exercise between trauma defined GWI groups and controls for the Miami cohort

Measure (units)	Mean ± SEM			*P*-value			g		
	HC	GWI_L	GWI_H	HC/GWI_L	HC/GWI_H	GWI_L/GWI_H	HC/GWI_L	HC/GWI_H	GWI_L/GWI_H
hIL-15 (a.u.)	0.30 ± 0.04	0.17 ± 0.01	0.29 ± 0.04	$4.89\times10^{-3*}$	9.88×10^{-1}	$3.76\times10^{-2*}$	0.66	0.04	0.59
BA (%)	0.40 ± 0.07	0.15 ± 0.03	0.19 ± 0.03	$9.99\times10^{-3*}$	$4.00\times10^{-2*}$	7.54×10^{-1}	0.60	0.52	0.19
HCT (%)	47.23 ± 0.58	45.76 ± 0.87	44.88 ± 0.48	4.59×10^{-1}	$1.58\times10^{-2*}$	7.55×10^{-1}	0.36	0.64	0.22

NK (%)	12.98 ± 1.09	8.76 ± 0.87	9.82 ± 1.13	$2.41\times10^{-2*}$	2.17×10^{-1}	8.39×10^{-1}	0.63	0.42	0.16
$CD3^{-}CD56^{+}$ (%)	24.17 ± 1.52	17.13 ± 0.92	20.73 ± 1.40	$2.32\times10^{-3*}$	3.01×10^{-1}	2.06×10^{-1}	0.77	0.34	0.43
$CD3^{-}CD56^{+}$ ($\times10^{9}$/L)$^{\#}$	771.27 ± 64.26	539.50 ± 46.67	606.81 ± 46.30	$2.88\times10^{-2*}$	1.42×10^{-1}	6.94×10^{-1}	0.59	0.34	0.22
$CD3^{-}CD16^{+}$ (%)	26.45 ± 1.60	18.79 ± 1.36	22.73 ± 1.67	$6.75\times10^{-3*}$	3.31×10^{-1}	3.16×10^{-1}	0.77	0.40	0.38
$CD3^{-}CD16^{+}$ ($\times10^{9}$/L)$^{\#}$	843.25 ± 68.32	592.56 ± 58.15	671.23 ± 55.79	$4.34\times10^{-2*}$	1.86×10^{-1}	7.18×10^{-1}	0.59	0.41	0.21
$CD2^{+}CD26^{+}$ (%)	35.94 ± 1.58	49.12 ± 3.05	39.64 ± 1.98	$1.32\times10^{-2*}$	4.15×10^{-1}	1.13×10^{-1}	1.04	0.31	0.64
$CD8^{+}CD26^{+}$ (%)	8.38 ± 0.70	15.92 ± 2.47	11.45 ± 1.45	7.5×10^{-2}	2.59×10^{-1}	4.42×10^{-1}	0.92	0.42	0.39
$CD3^{-}CD16^{+}CD11a^{+}$ (%)	26.34 ± 1.72	18.61 ± 1.39	23.11 ± 1.72	$9.00\times10^{-3*}$	4.67×10^{-1}	2.46×10^{-1}	0.73	0.28	0.42

g corrected hedge's g effect size, *GWI* Gulf War Illness, GWI_H Gulf War Illness with high probability of PTSD symptoms, GWI_L Gulf War Illness with low probability of PTSD symptoms, *HC* healthy control, *P* pairwise significance measure as determined by a Games-Howell post-hoc analysis, *SEM* standard error of the mean. $^{*}P < 0.05$

Table S6 Welch ANOVA Omnibus significant comparisons of hormones, cytokines, complete blood count and flow cytometry measures at 4h post exercise between trauma defined GWI groups and controls for the Miami cohort

Measure (units)	Mean ± SEM			*P*-value			g		
	HC	GWI_L	GWI_H	HC/GWI_L	HC/GWI_H	GWI_L/GWI_H	HC/GWI_L	HC/GWI_H	GWI_L/GWI_H
hIL-15 (a.u.)	0.22 ± 0.02	0.14 ± 0	0.22 ± 0	$4.42\times10^{-2*}$	9.99×10^{-1}	2.15×10^{-1}	0.52	0.01	0.40

g corrected hedge's g effect size, *GWI* Gulf War Illness, GWI_H Gulf War Illness with high probability of PTSD symptoms, GWI_L Gulf War Illness with low probability of PTSD symptoms, *HC* healthy control, *P* pairwise significance measure as determined by a Games-Howell post-hoc analysis, *SEM* standard error of the mean. $^{*}P < 0.05$

Table S7 Graph edit distance (GED) comparison between GWI subgroups and controls across time

Timepoint	HC & GWI_L	HC & GWI_H	GWI_L & GWI_H
T0	127.91	111.84	107.40
T1	117.53	116.93	118.88
T2	97.19	100.80	86.07

GWI Gulf War Illness, GWI_H Gulf War Illness with high probability of PTSD symptoms, GWI_L Gulf War Illness with low probability of PTSD symptoms, *HC* healthy control, *T0* timepoint 0 – at rest, *T1* timepoint 1 – at peak exercise, *T2* timepoint 2 – 4 h after peak exercise

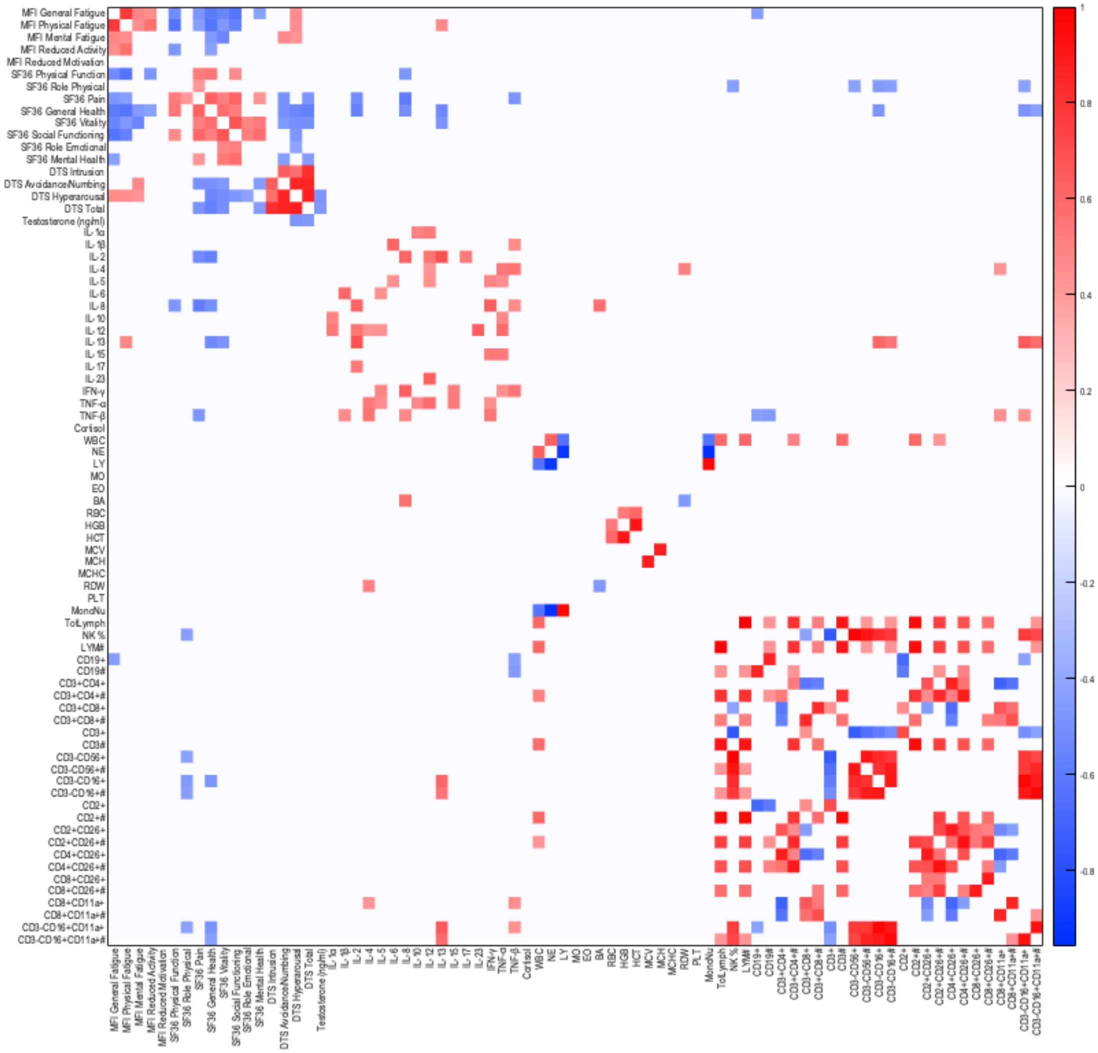

Fig. S1 Correlation graph for HC at rest. Spearman correlation ρ values range between -1 and 1 as indicated in the colormap on the right. All correlations shown have been corrected for multiple comparisons with a q-value < 0.05. HC healthy control

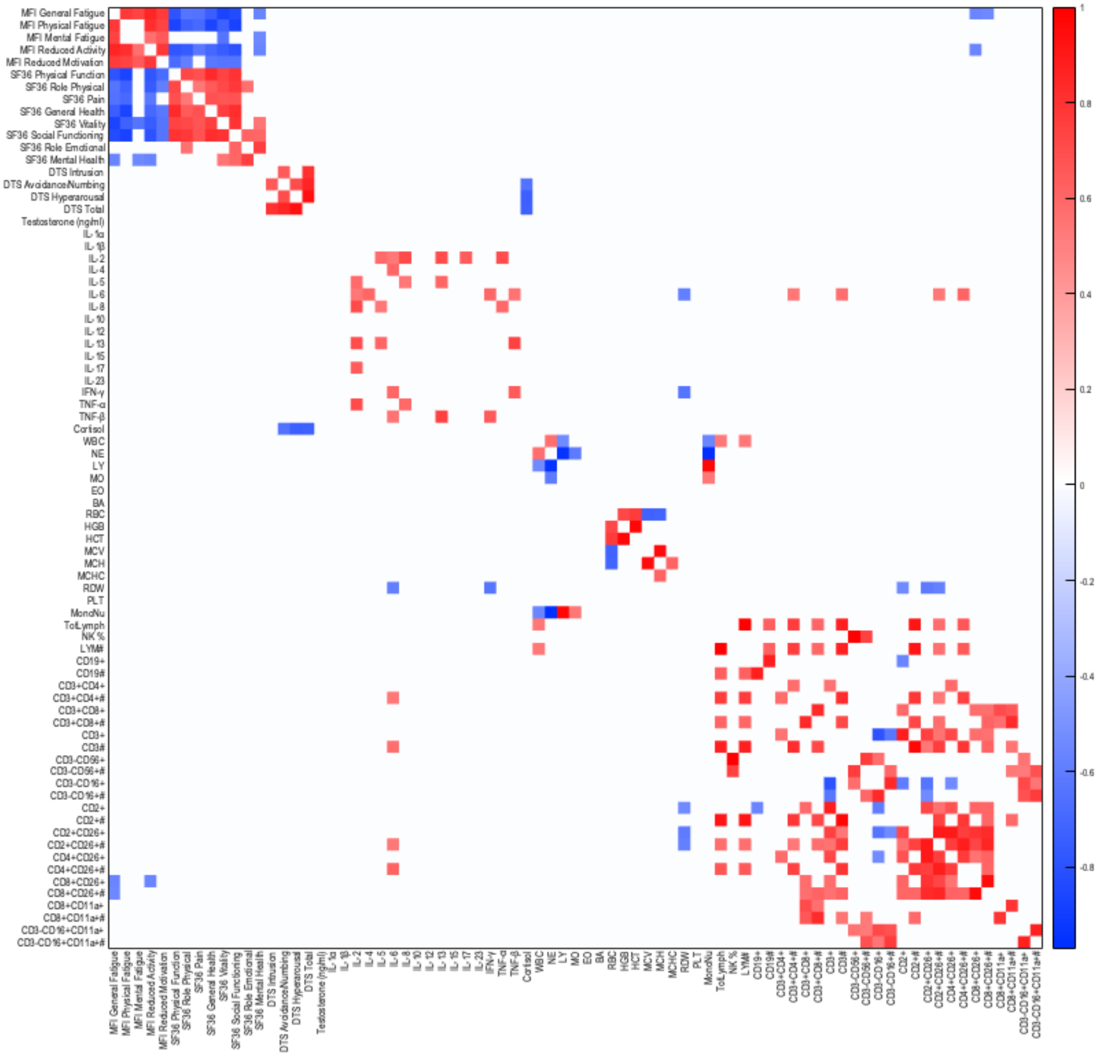

Fig. S2 Correlation graph for GWI_L at rest. Spearman correlation ρ values range between -1 and 1 as indicated in the colormap on the right. All correlations shown have been corrected for multiple comparisons with a *q*-value < 0.05. GWI_L Gulf War illness with low probability of PTSD symptoms

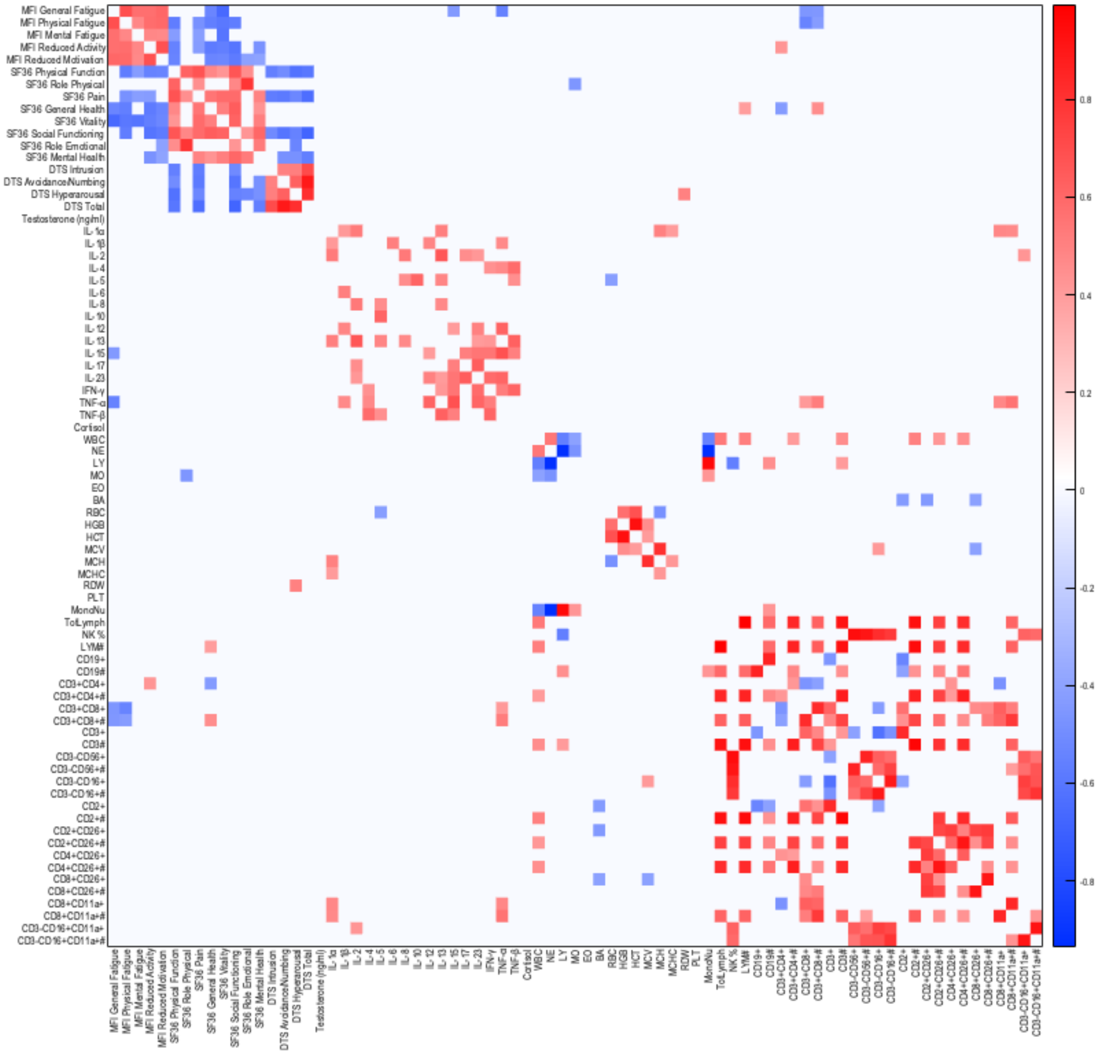

Fig. S3 Correlation graph for GWI_H at rest. Spearman correlation ρ values range between -1 and 1 as indicated in the colormap on the right. All correlations shown have been corrected for multiple comparisons with a *q*-value < 0.05. GWI_H Gulf War illness with high probability of PTSD symptoms

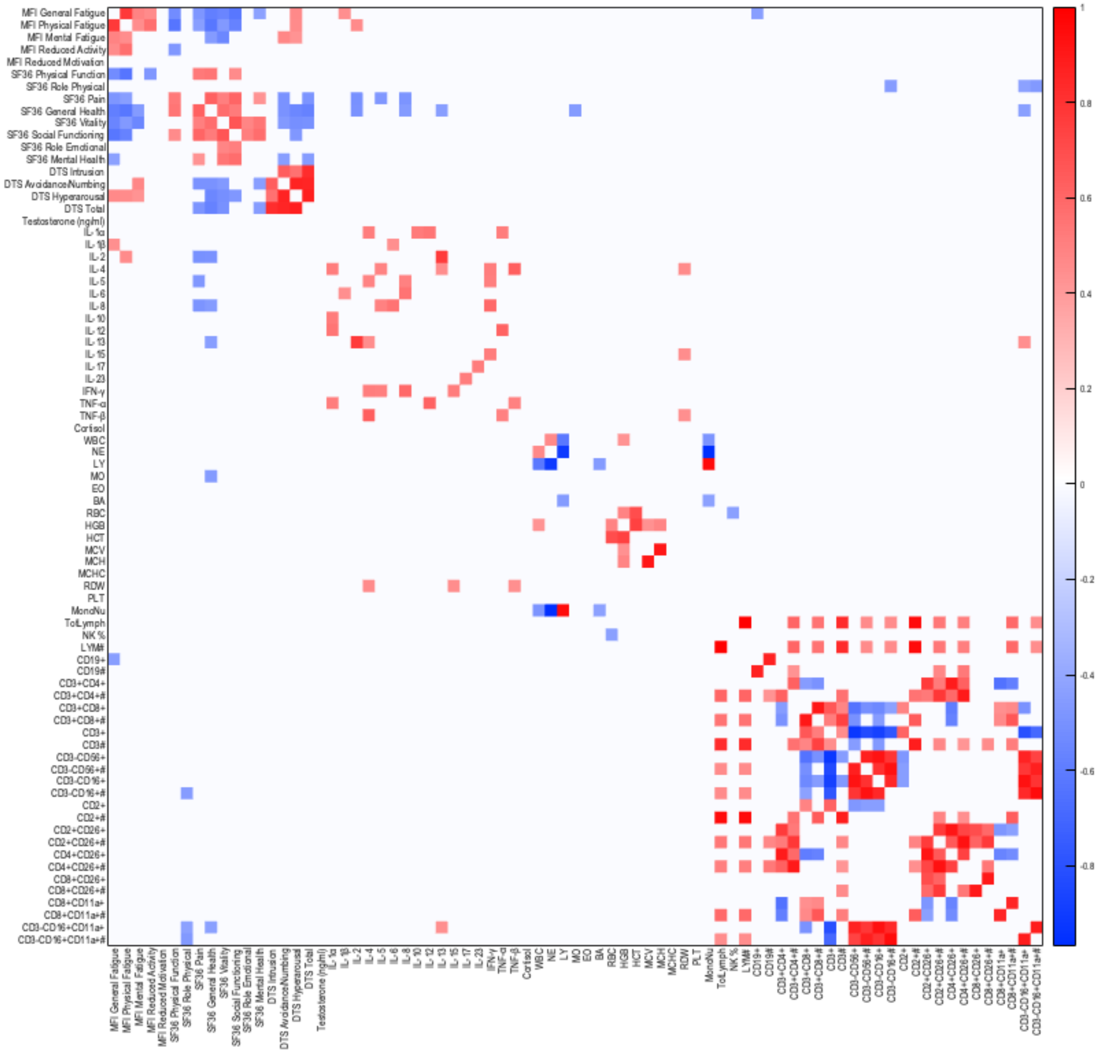

Fig. S4 Correlation graph for HC at peak exercise. Spearman correlation ρ values range between -1 and 1 as indicated in the colormap on the right. All correlations shown have been corrected for multiple comparisons with a *q*-value < 0.05. HC healthy control

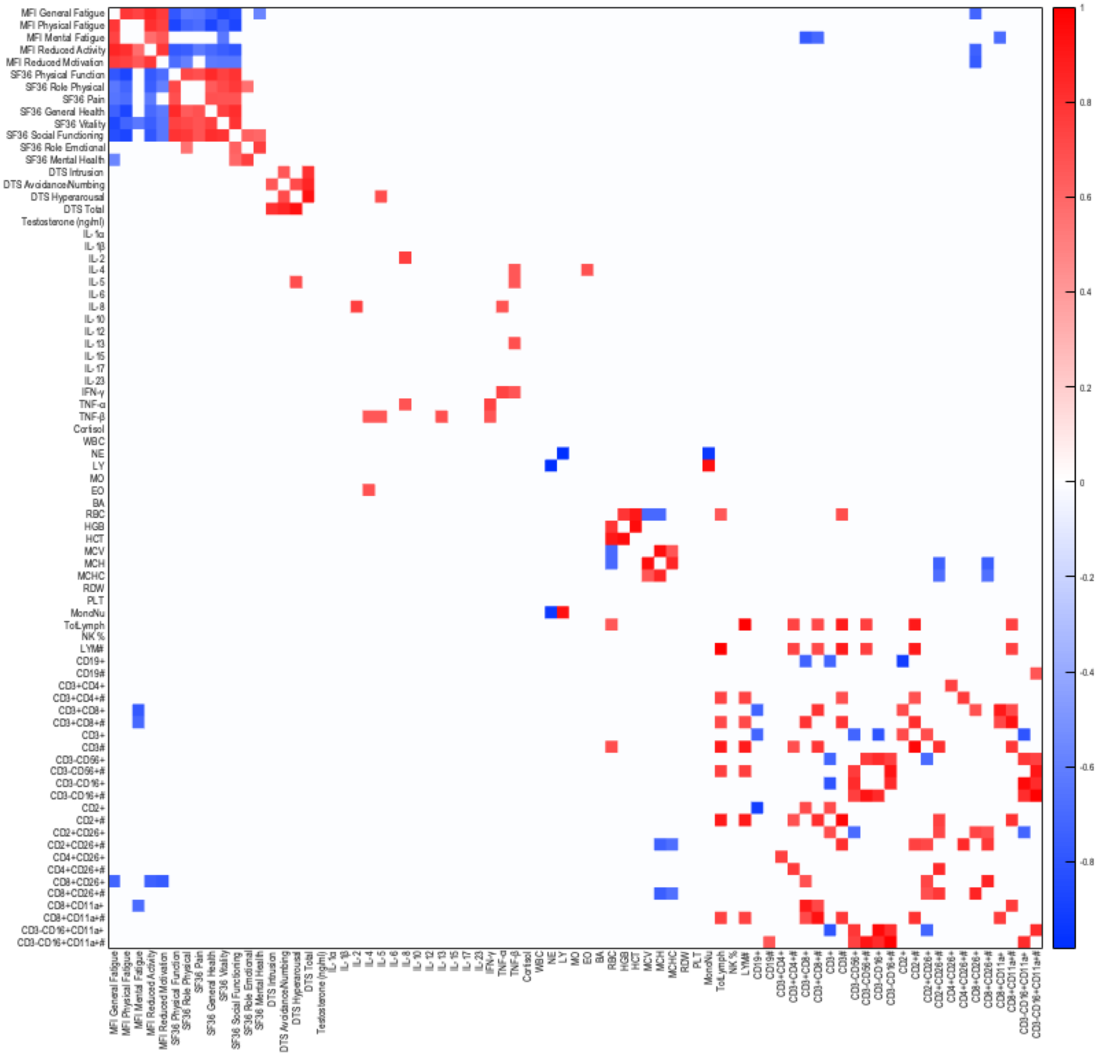

Fig. S5 Correlation graph for GWI_L at peak exercise. Spearman correlation ρ values range between -1 and 1 as indicated in the colormap on the right. All correlations shown have been corrected for multiple comparisons with a *q*-value < 0.05. GWI_L Gulf War illness with low probability of PTSD symptoms

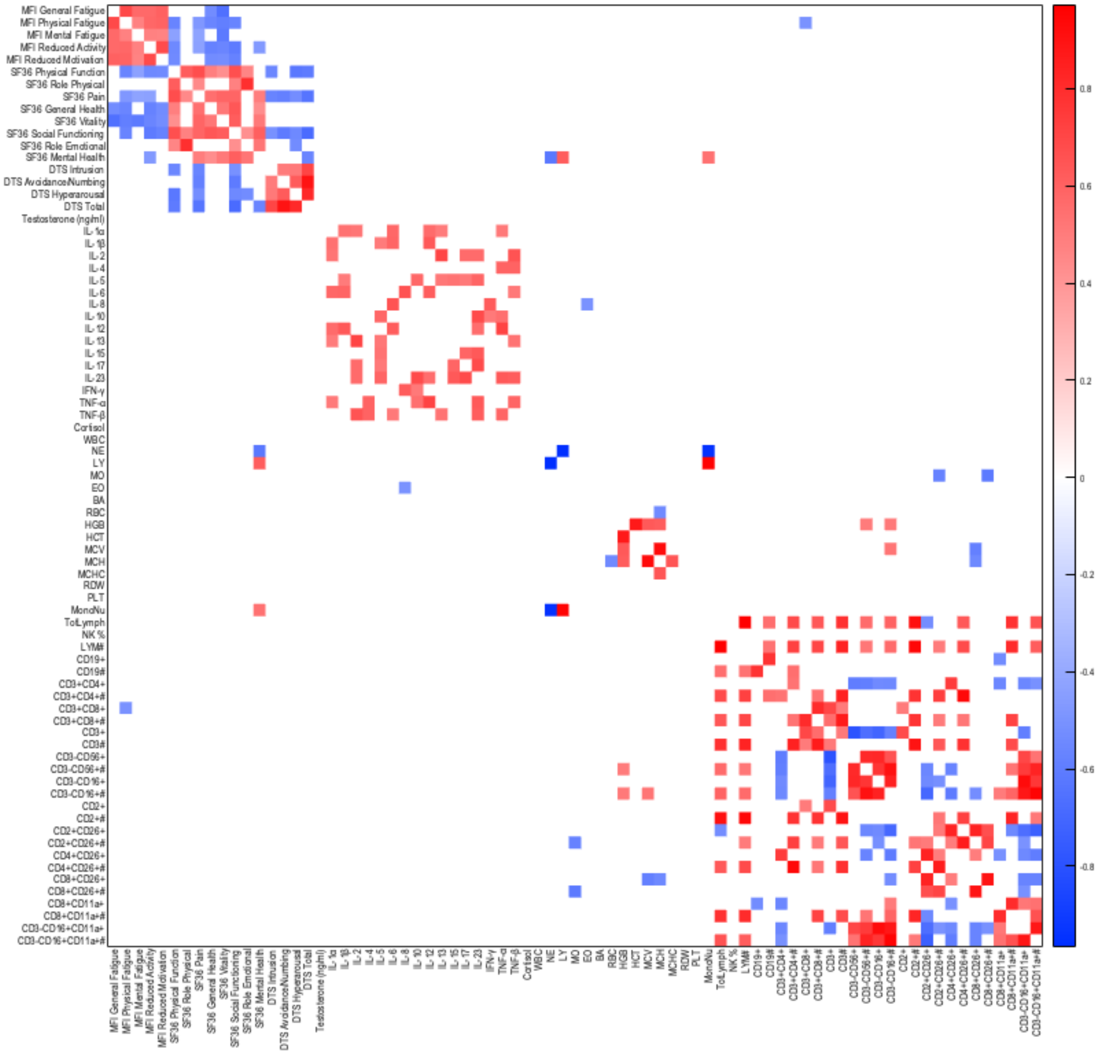

Fig. S6 Correlation graph for GWI_H at peak exercise. Spearman correlation ρ values range between -1 and 1 as indicated in the colormap on the right. All correlations shown have been corrected for multiple comparisons with a *q*-value < 0.05. GWI_H Gulf War illness with high probability of PTSD symptoms

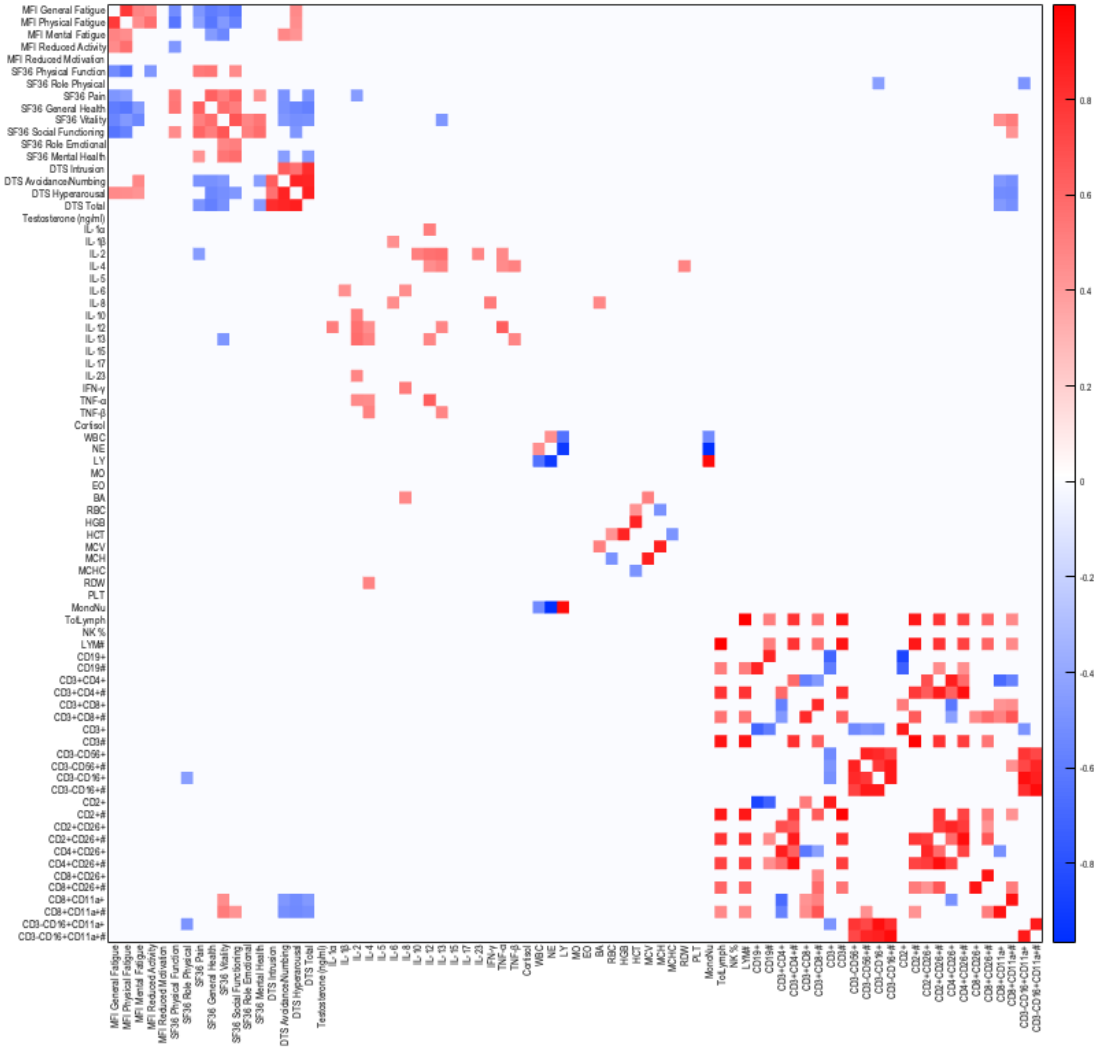

Fig. S7 Correlation graph for HC four hours post exercise. Spearman correlation ρ values range between -1 and 1 as indicated in the colormap on the right. All correlations shown have been corrected for multiple comparisons with a *q*-value < 0.05. HC healthy control

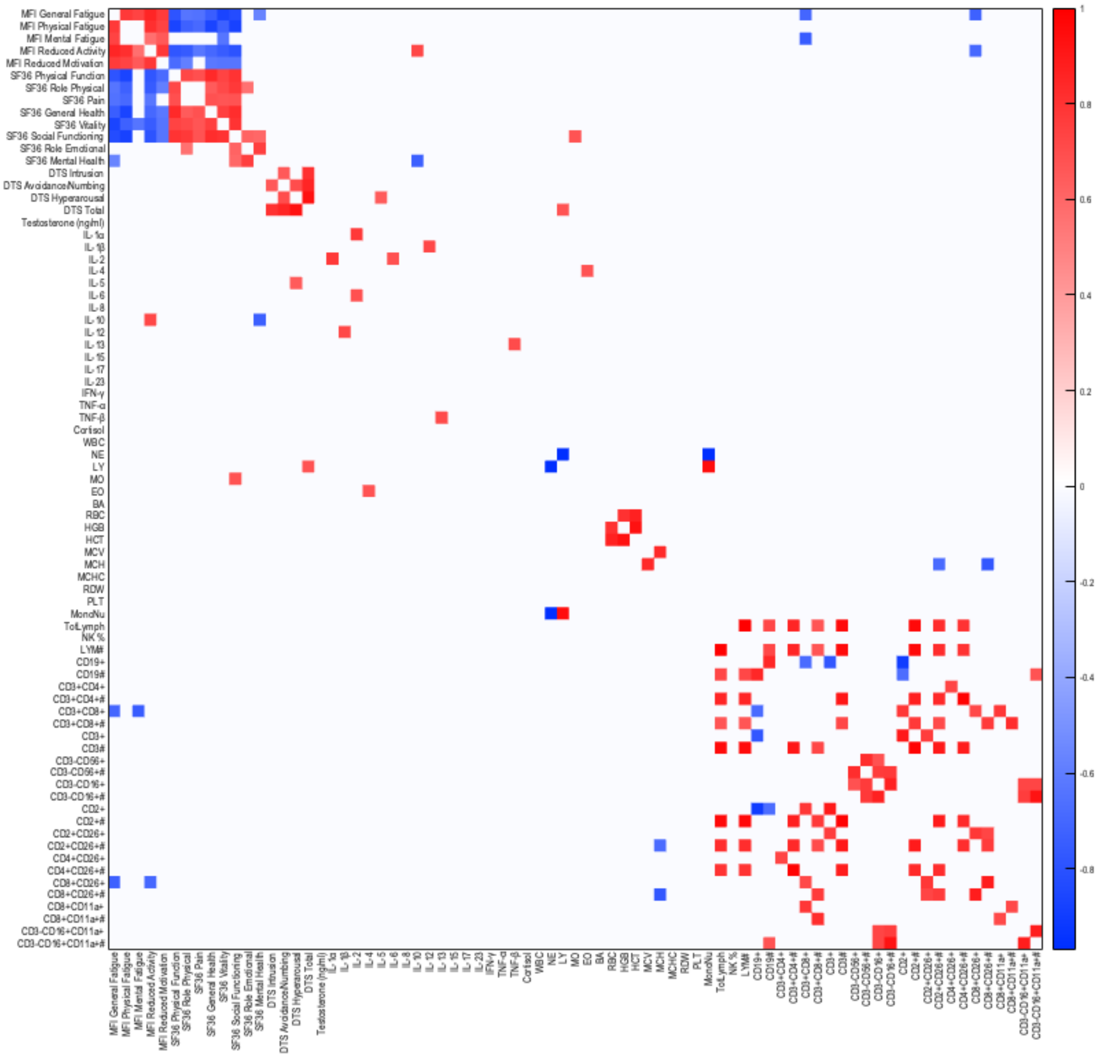

Fig. S8 Correlation graph for GWI_L four hours post exercise. Spearman correlation ρ values range between -1 and 1 as indicated in the colormap on the right. All correlations shown have been corrected for multiple comparisons with a *q*-value < 0.05. GWI_L Gulf War illness with low probability of PTSD symptoms

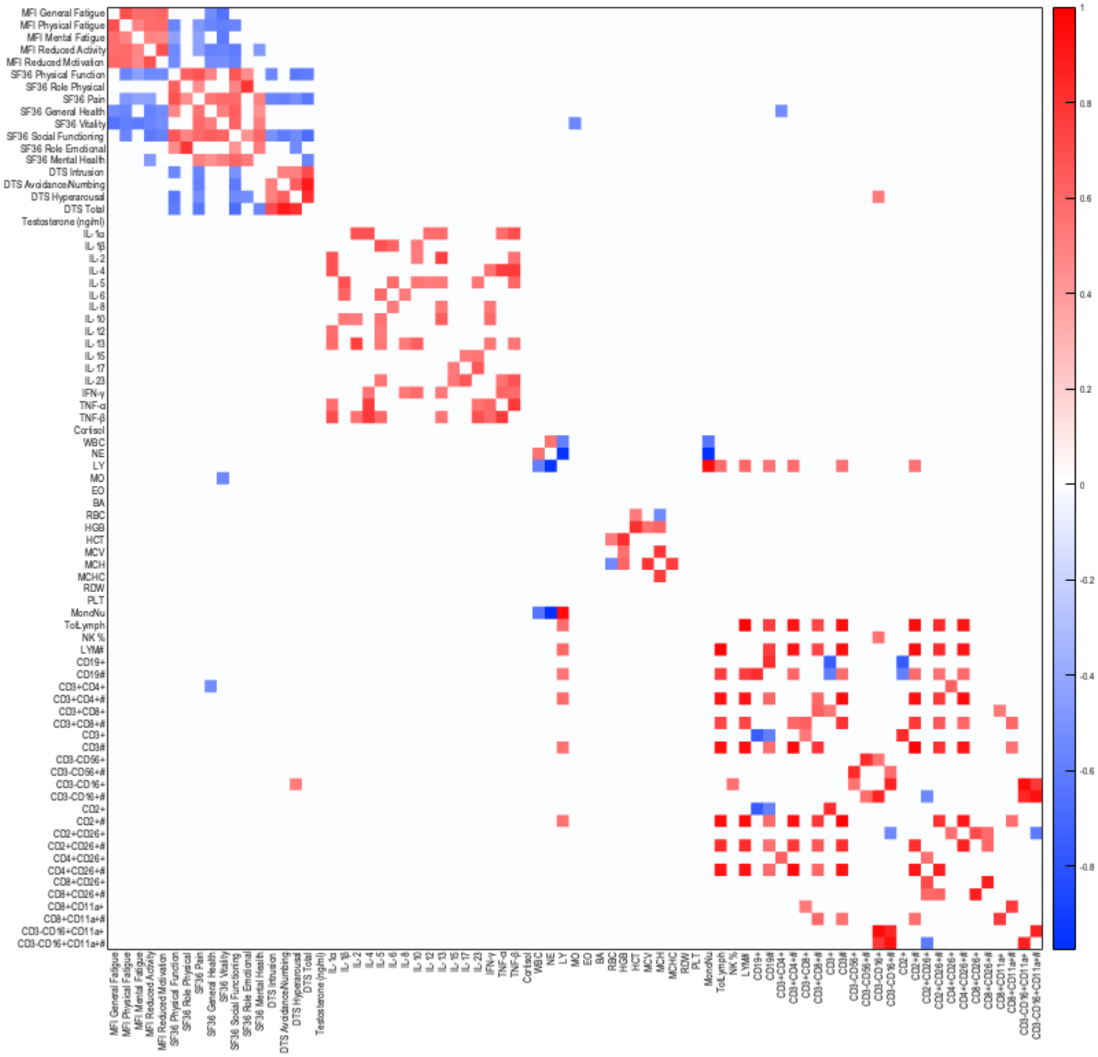

Fig. S9 Correlation graph for GWI_H four hours post exercise. Spearman correlation ρ values range between -1 and 1 as indicated in the colormap on the right. All correlations shown have been corrected for multiple comparisons with a *q*-value < 0.05. GWI_H Gulf War illness with high probability of PTSD symptoms
