# Supplementary material for: Disentangling the effects of PTSD from Gulf War Illness in male veterans via a systems-wide analysis of immune cell, cytokine, and symptom measures
Source: Mil Med Res. 2024 Jan 2;11:2. doi: 10.1186/s40779-023-00505-4 (PMC10759613; doi:10.1186/s40779-023-00505-4)
Supplement: Supplementary file 2 — Additional file 2. Flow cytometry gating strategy. [file 40779_2023_505_MOESM2_ESM.pdf]

Institution: VAMC-Univ. of Miami Clinical Immunology

Protocol: CD45-4-19-14.PRO

Runtime Results

Analysis Date: 30-Oct-2012, 11:26:28

Settings File: WASHED VAMC 4C1L settings.pro, 30-Oct-2012, 10:56:15

Listmode File: 270390 00055112 1126 001.LMD

FCS Information
270390

Run Date: 30-Oct-12, 11:26:14

Sample ID: 270390

User ID: ZENG

Acquisition Time/Events: 13.1s / 6452 (MANUAL)

Tube ID: NoRead

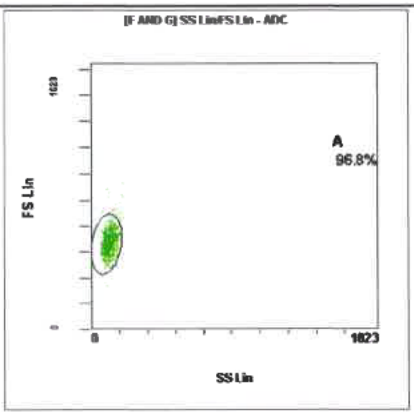

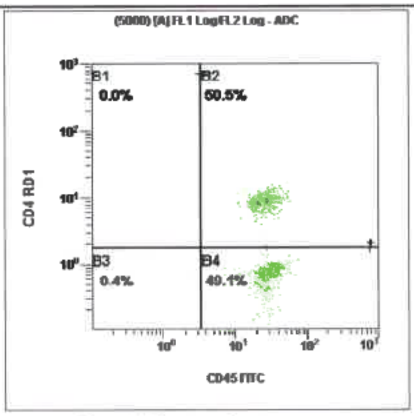

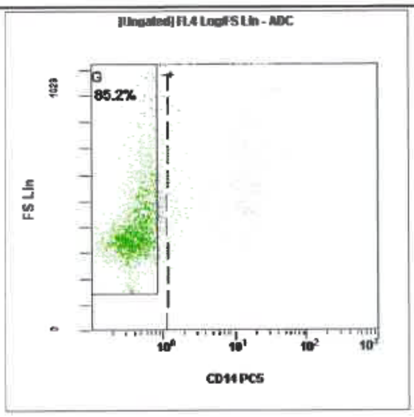

(5000) [A] FL1 Log/FL2 Log

Region	Number	%Gated
ALL	2543	100.000
B1	0	0.000
B2	1285	50.531
B3	9	0.354
B4	1249	49.115

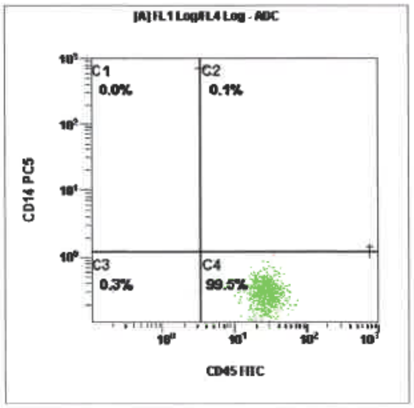

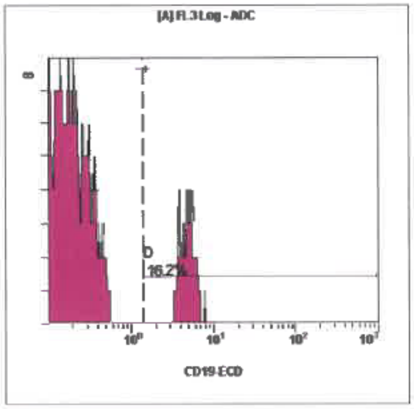

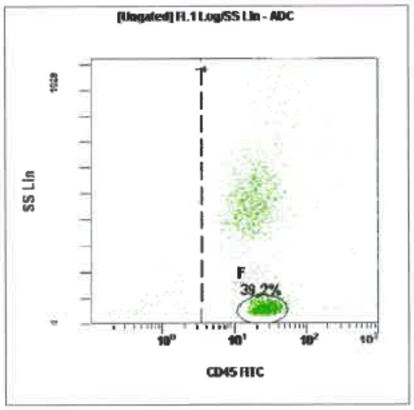

[A] FL1 Log/FL4 Log

Region	Number	%Gated
ALL	2543	100.000
C1	1	0.039
C2	3	0.118
C3	8	0.315
C4	2531	99.528

[A] FL3 Log

Region	Number	%Gated
ALL	2543	100.000
D	413	16.241

Plot/Legend not found

[Ungated] FL1 Log/SS Lin

Region	Number	%Gated
ALL	6452	100.000
F	2529	39.197

Institution: VAMC-Univ. of Miami Clinical Immunology

Protocol: ISOTYPE.PRO

Runtime Results

Analysis Date: 30-Oct-2012, 11:27:07

Settings File: WASHED VAMC 4C1L settings.pro, 30-Oct-2012, 10:56:15

Listmode File: 270390 00055113 1127 002.LMD

FCS Information
270390

Run Date: 30-Oct-12, 11:26:55

Sample ID: 270390

User ID: ZENG

Acquisition Time/Events: 11.2s / 4727 (MANUAL)

Tube ID: NoRead

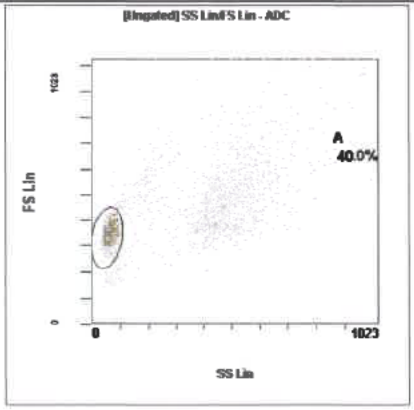

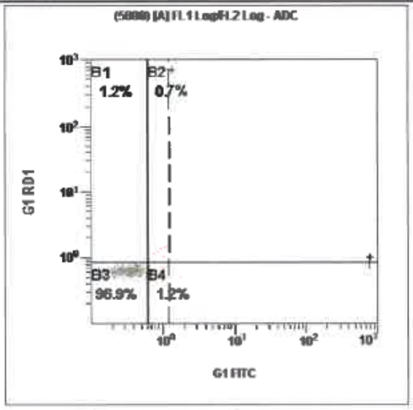

(5000) [A] FL1 Log/FL2 Log

Region	Number	%Gated
ALL	1893	100.000
B1	22	1.162
B2	14	0.740
B3	1835	96.936
B4	22	1.162

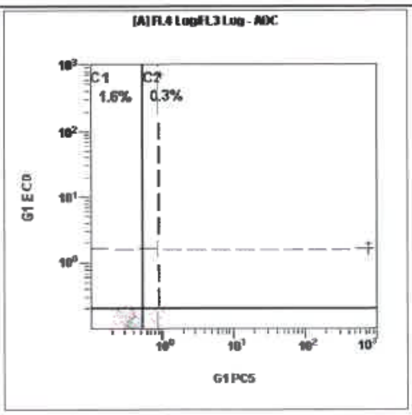

[A] FL4 Log/FL3 Log

Region	Number	%Gated
ALL	1893	100.000
C1	31	1.638
C2	5	0.264
C3	1825	96.408
C4	32	1.690

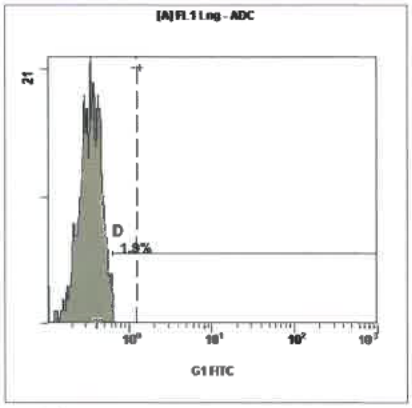

[A] FL1 Log

Region	Number	%Gated
ALL	1893	100.000
D	36	1.902

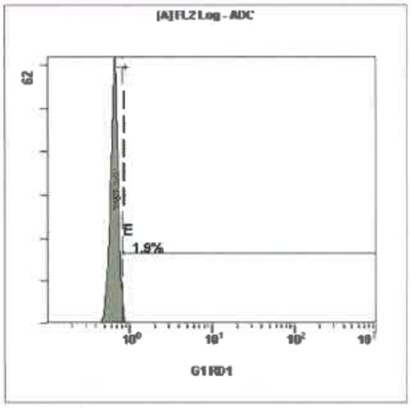

[A] FL2 Log

Region	Number	%Gated
ALL	1893	100.000
E	36	1.902

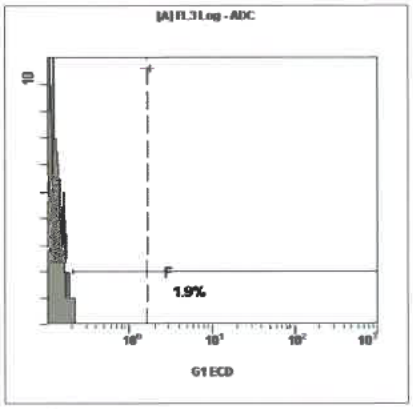

[A] FL3 Log

Region	Number	%Gated
ALL	1893	100.000
F	36	1.902

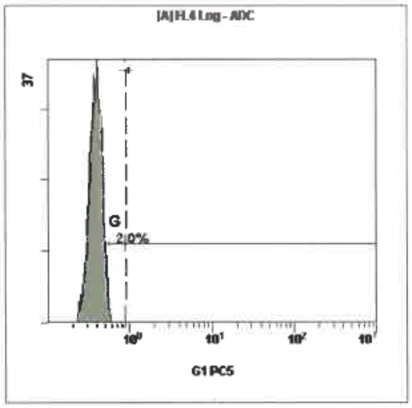

[A] FL4 Log

Region	Number	%Gated
ALL	1893	100.000
G	37	1.955

Institution: VAMC-Univ. of Miami Clinical Immunology

Protocol: TB StudyTUBE 2.PRO

Runtime Results

Analysis Date: 30-Oct-2012, 11:28:00

Settings File: WASHED VAMC 4C1L settings.pro, 30-Oct-2012, 10:56:15

Listmode File: 270390 00055114 1128 003.LMD

FCS Information
270390

Run Date: 30-Oct-12, 11:27:35

Sample ID: 270390

User ID: ZENG

Acquisition Time/Events: 24.7s / 5494 (MANUAL)

Tube ID: NoRead

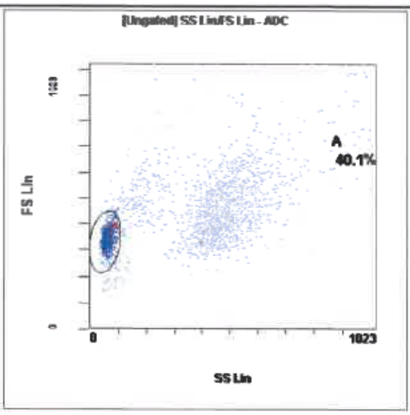

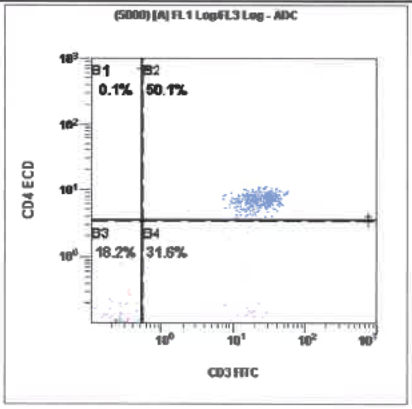

(5000) [A] FL1 Log/FL3 Log

Region	Number	%Gated
ALL	2205	100.000
B1	2	0.091
B2	1105	50.113
B3	402	18.231
B4	696	31.565

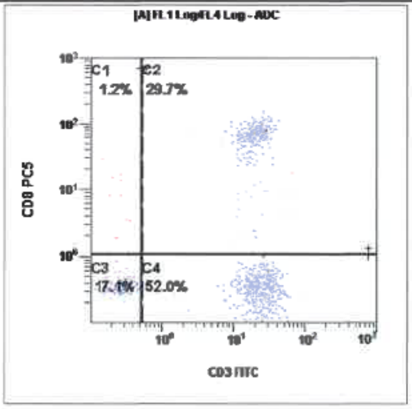

[A] FL1 Log/FL4 Log

Region	Number	%Gated
ALL	2205	100.000
C1	26	1.179
C2	654	29.660
C3	378	17.143
C4	1147	52.018

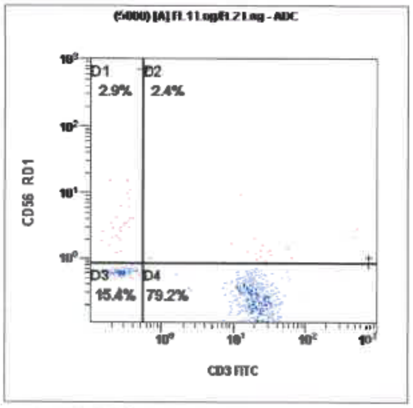

(5000) [A] FL1 Log/FL2 Log

Region	Number	%Gated
ALL	2205	100.000
D1	65	2.948
D2	53	2.404
D3	340	15.420
D4	1747	79.229

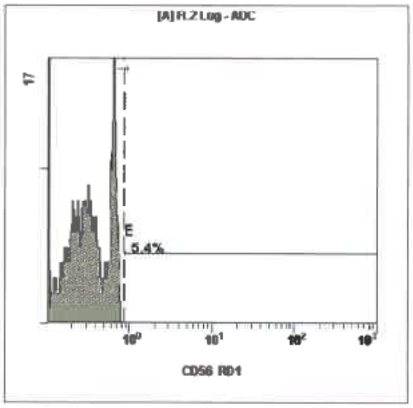

[A] FL2 Log

Region	Number	%Gated
ALL	2205	100.000
E	118	5.351

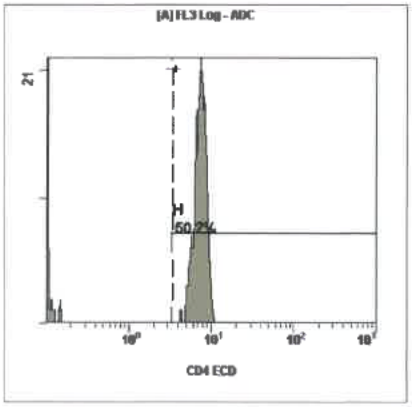

[A] FL3 Log

Region	Number	%Gated
ALL	2205	100.000
H	1107	50.204

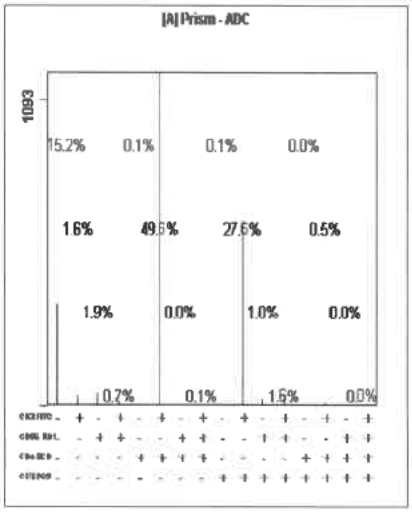

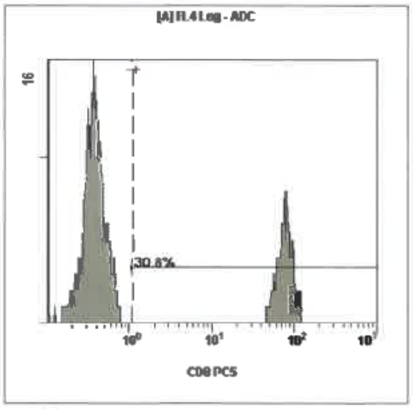

[A] FL4 Log

Region	Number	%Gated
ALL	2205	100.000
I	680	30.839

Institution: VAMC-Univ. of Miami Clinical Immunology

Protocol: CD2-26-4-8.PRO

Runtime Results

Analysis Date: 30-Oct-2012, 11:29:03

Settings File: WASHED VAMC 4C1L settings.pro, 30-Oct-2012, 10:56:15

Listmode File: 270390 00055115 1129 004.LMD

Run Date: 30-Oct-12, 11:28:33

Sample ID: 270390

User ID: ZENG

Acquisition Time/Events: 28.7s / 5786 (MANUAL)

Tube ID: NoRead

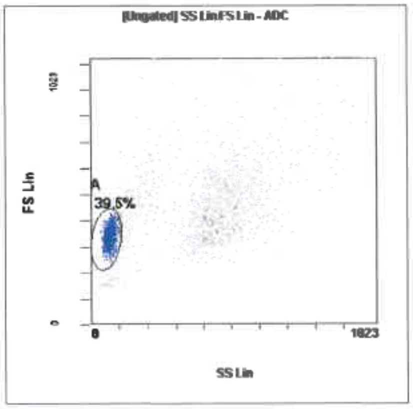

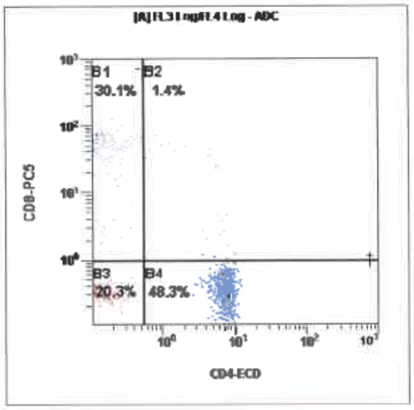

[A] FL3 Log/FL4 Log

Region	Number	%Gated	X-Median 50.0	Y-Median 50.0
ALL	2284	100.00	0.333	0.424
B1	687	30.08	0.102	57.1
B2	31	1.36	4.4	14
B3	463	20.27	0.102	0.354
B4	1103	48.29	7.28	0.324

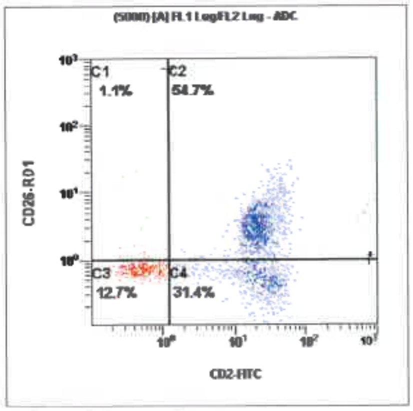

(5000) [A] FL1 Log/FL2 Log

Region	Number	%Gated	X-Median 50.0	Y-Median 5(
ALL	2284	100.00	20.3	1.53
C1	26	1.14	0.472	1.38
C2	1249	54.68	22	3.36
C3	291	12.74	0.503	0.728
C4	718	31.44	23.4	0.546

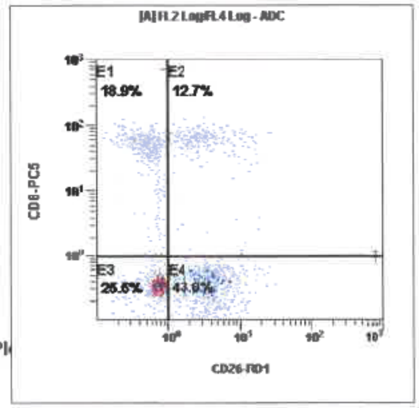

Pl

[A] FL2 Log/FL4 Log

Region	Number	%Gated	X-Median 50.0	Y-Median 50.0
ALL	2284	100.00	1.53	0.424
E1	431	18.87	0.508	51.7
E2	289	12.65	2.66	64.7
E3	583	25.53	0.683	0.351
E4	981	42.95	3.51	0.324

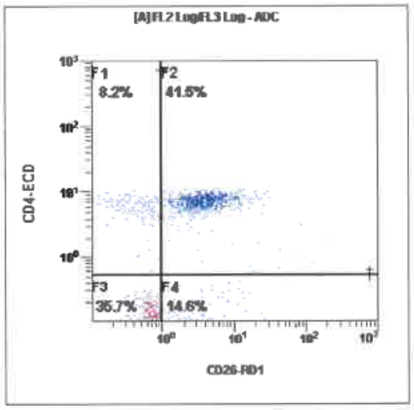

[A] FL2 Log/FL3 Log

Region	Number	%Gated	X-Median 50.0	Y-Median 50.0
ALL	2284	100.00	1.53	0.333
F1	187	8.19	0.444	6.71
F2	947	41.46	3.57	7.41
F3	816	35.73	0.641	0.102
F4	334	14.62	2.59	0.102

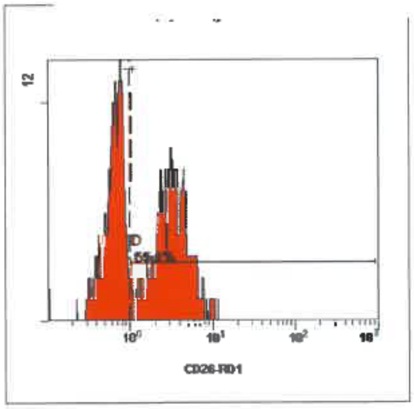

dian 50.0	Y-Median 50.0
	###
	###

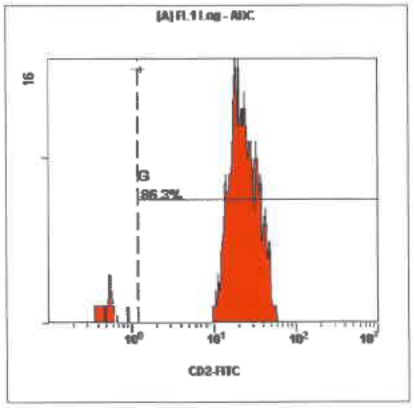

[A] FL1 Log

Region	Number	%Gated	X-Median 50.0	Y-Median 50.0
ALL	2284	100.00	20.3	###
G	1971	86.30	22.4	###

Institution: VAMC-Univ. of Miami Clinical Immunology

Protocol: 16-11a-8-3.PRO

Runtime Results

Analysis Date: 30-Oct-2012, 11:30:43

Settings File: WASHED VAMC 4C1L settings.pro, 30-Oct-2012, 10:56:15

Listmode File: 270390 00055116 1130 005.LMD

FCS Information
270390

Run Date: 30-Oct-12, 11:29:36

Sample ID: 270390

User ID: ZENG

Acquisition Time/Events: 66.0s / 5444 (MANUAL)

Tube ID: NoRead

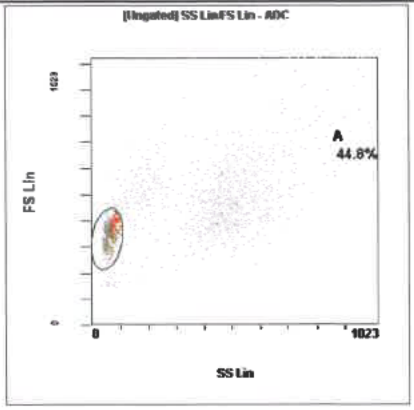

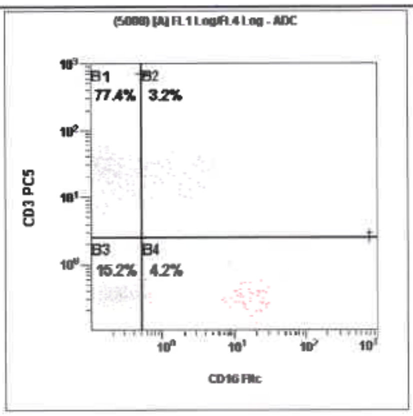

(5000) [A] FL1 Log/FL4 Log

Region	Number	%Gated
ALL	2439	100.000
B1	1888	77.409
B2	79	3.239
B3	370	15.170
B4	102	4.182

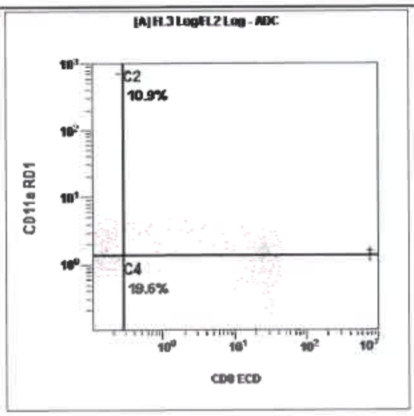

[A] FL3 Log/FL2 Log

Region	Number	%Gated
ALL	2439	100.000
C1	1017	41.697
C2	265	10.865
C3	678	27.798
C4	479	19.639

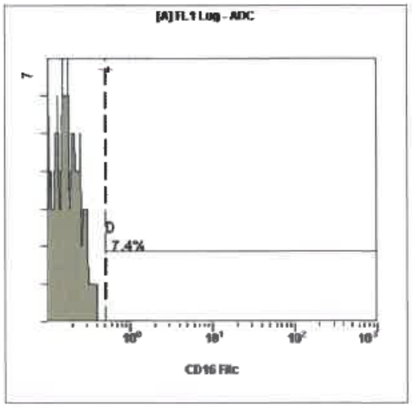

[A] FL1 Log

Region	Number	%Gated
ALL	2439	100.000
D	181	7.421

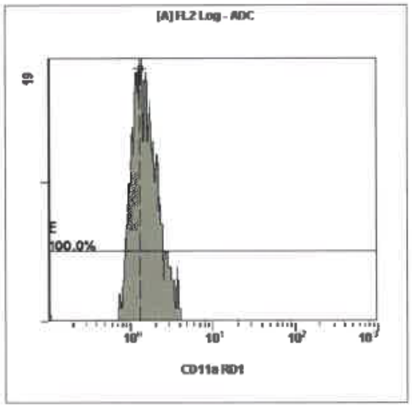

[A] FL2 Log

Region	Number	%Gated
ALL	2439	100.000
E	2439	100.000

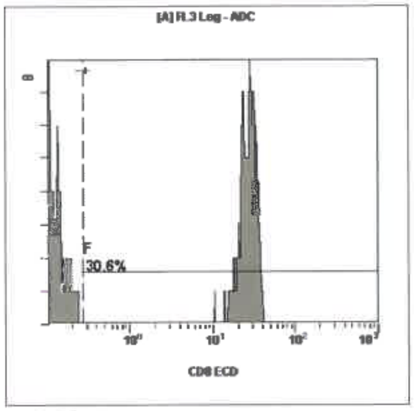

[A] FL3 Log

Region	Number	%Gated
ALL	2439	100.000
F	747	30.627

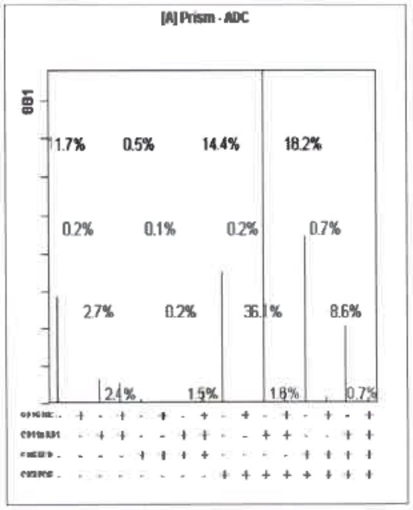

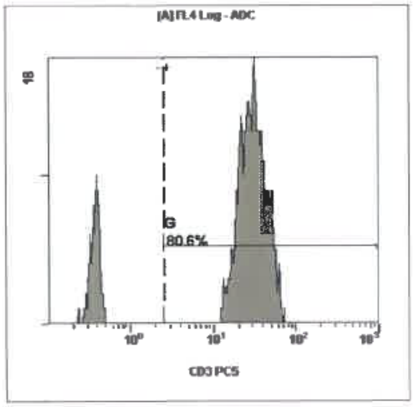

[A] FL4 Log

Region	Number	%Gated
ALL	2439	100.000
G	1967	80.648

CXP Panel Report:

VAMC-Univ. of Miami Clinical Immunology

10/30/2012 11:30:45AM Runtime Results

Sample ID1: 270390
Panel Name: GULF WAR

Name:
Patient ID: [redacted]
D.O.B.:

Panel Complete: Y Match: Y
LMD File Name(s):
C:\CXP\Users\Flow\LMD\270390 00055112 1126 001.LMD
C:\CXP\Users\Flow\LMD\270390 00055116 1130 005.LMD
Collection Date: [redacted]
Analysis Date / Time:
Sex: ID#:
Physician: KLIMAS
Sample Type: Whole Blood
Dilution Factor:
Harvest Volume:
Body Weight:

FC 500 SN: AH05013
CXP v 2.2:

User ID: ZENG
Tube ID: NoRead
Hematology Date /Time: [redacted]
Hematology Instrument: HMX
WBC: 5.50x10^3/uL
RBC: 4.660x10^6/uL
PLT: 220.0x10^3/uL

LY %: 38.30
MO %: 6.30
NE %: 51.60
EO %: 2.90
BA %: 0.90

Description	Region	Cell Pop	Result	Cells/uL HEM	Opt Stat 1	Opt Stat 2
Total B Cells (CD19+)	eq		16.318%	344.0		
**Expected Range**			8.000 - 18.000	128 - 400		
T-Helper (CD3+CD4+)	eq		50.351%	1061.0		
**Expected Range**			38.000 - 53.000	595 - 1199		
T-Cytotoxic/Suppressor CD3+CD8+	eq		29.800%	628.0		
**Expected Range**			15.000 - 32.000	219 - 731		
T Cells (CD3+)	eq		82.065% H	1729.0		
**Expected Range**			66.000 - 80.000	989 - 1899		
Natural Killer Cells CD3-CD56+	eq		2.962% L	62.0 L		
**Expected Range**			5.000 - 16.000	98 - 294		
CD3-CD16+	eq		4.202%	89.0		
**Expected Range**						
Total CD2+	eq		86.529% H	1823.0		
**Expected Range**			75.000 - 86.000	1130 - 1985		
CD2+CD26+	eq		54.944%	1157.0		
**Expected Range**			38.000 - 60.000	593 - 1375		
CD4+CD26+	eq		41.659%	878.0		
**Expected Range**						
CD8+CD26+	eq		12.713%	268.0		
**Expected Range**						
CD8+CD11a+	eq		10.917%	230.0		
**Expected Range**			8.000 - 23.000	139 - 472		
CD3-CD16+CD11a+	eq		6.344%	134.0		

Signature X Zeng

10/30/2012 11:30:45AM Runtime Results

Sample ID1: 270390

Name:

Panel Name: GULF WAR

Patient ID: [redacted]

D.O.B.:

Description	Region	Cell Pop	Result	Cells/uL HEM	Opt Stat 1	Opt Stat 2
Expected Range						

Signature ____________________

CXP Panel Report:

VAMC-Univ. of Miami Clinical Immunology

10/30/2012 11:30:45AM Runtime Results

Sample ID1: 270390

Panel Name: GULF WAR

Name:

Patient ID: [redacted]

D.O.B.:

Description	Region	Cell Pop	Result	Cells/uL HEM	Opt Stat 1	Opt Stat 2
Recovery	A	LY	96.823%	2040.0		
Expected Range						
Purity	C4	LY	99.528%	2097.0		
Expected Range						
Total T-Cells	eq		82.065%	1729.0		
Expected Range						
(CD3+CD4+) + (CD3+CD8+)	eq		80.151%	1688.0		
Expected Range						
Lymphosum (T+B+NK)	eq		101.345%	2135.0		
Expected Range						
Total CD4	eq		50.770%	1069.0		
Expected Range						
Total CD4	eq		50.351%	1061.0		
Expected Range						
Total CD4	eq		49.885%	1051.0		
Expected Range						
Total CD8	eq		30.985%	653.0		
Expected Range						
Total CD8	eq		31.585%	665.0		
Expected Range						
Total CD8	eq		30.773%	648.0		
Expected Range						

Comments:

Signature ____________________
